# Ni-promoted reductive cyclization cascade enables a total synthesis of (+)-aglacin B

**DOI:** 10.3762/bjoc.21.197

**Published:** 2025-11-18

**Authors:** Si-Chen Yao, Jing-Si Cao, Jian Xiao, Ya-Wen Wang, Yu Peng

**Affiliations:** 1 School of Chemistry, Southwest Jiaotong University, Chengdu 610031, People’s Republic of China; 2 School of Materials and Environment Engineering, Chengdu Technological University, Chengdu 611730, People’s Republic of China

**Keywords:** aryltetralin, conjugate addition, cyclolignan, nickel, reductive coupling

## Abstract

The total synthesis of bioactive (+)-aglacin B was achieved. The key steps include an asymmetric conjugate addition reaction induced by a chiral auxiliary and a nickel-promoted reductive tandem cyclization of the elaborated β-bromo acetal, which led to the efficient construction of the aryltetralin[2,3-*c*]furan skeleton embedded in this natural product.

## Introduction

**Figure 1:**
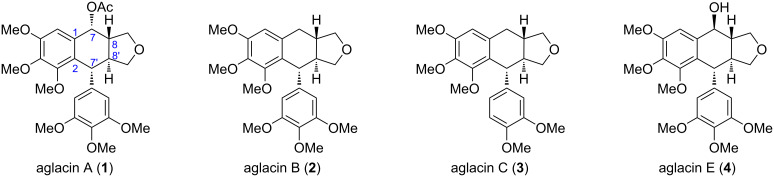
The structures of aglacins A, B, C, and E.

Proksch and co-workers isolated aglacins A, B, C, and E (**1**–**4**, Figure 1) from the methanolic extract of stem bark of *Aglaia cordata* Hiern from the tropical rain forests of the Kalimantan region (Indonesia) [1–2]. These cyclic ether natural products belong to the typical aryltetralin lignans, which have already attracted broad attention from the synthetic community [3–5]. Zhu and co-workers disclosed a concise synthesis of (±)-aglacins B (**2**) and C (**3**), featuring a visible light-catalyzed radical cation cascade for the formation of the C8–C8′ and C2–C7′ bonds [6]. Subsequently, they improved the reaction conditions to achieve the racemic synthesis of aglacins A (**1**) and E (**4**) as well [7]. In 2021, the Gao group described the total synthesis of both enantiomers of aglacins A (**1**), B (**2**), and E (**4**) by asymmetric photoenolization/Diels–Alder reactions as the key steps for the construction of the C7–C8 and C7′–C8′ bonds [8].

During the past decade, we had developed nickel-catalyzed or -promoted reductive coupling/cyclization reactions for the formation of inter- or intramolecular carbon–carbon bonds under mild conditions [9–12], and strategically applied this method for the divergent syntheses of some natural products [13–17]. Herein, we report our recent advance to a total synthesis of (+)-aglacin B (**2**), which relies on a non-photocatalysis approach.

## Results and Discussion

### Retrosynthetic analysis of (+)-aglacin B

**Scheme 1:**
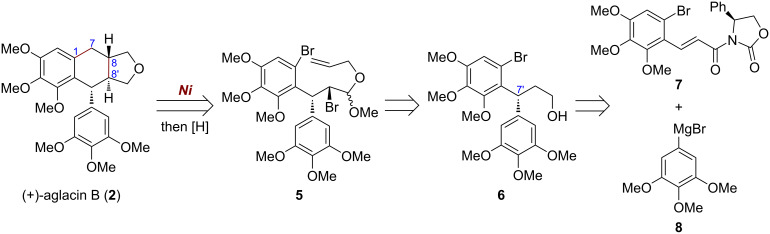
Retrosynthetic analysis of (+)-aglacin B (**2**).

Based on the retrosynthetic analysis shown in Scheme 1, both C8′–C8 and C7–C1 bonds in (+)-aglacin B (**2**) could be constructed in one-step from the β-bromo acetal **5** by a Ni-promoted tandem radical cyclization, and a subsequent acetal reduction under acidic conditions then can complete the total synthesis of this molecule. The cyclization precursor **5** could be prepared from the primary alcohol **6** through transforming functional groups of the alkyl chain and installing an allyl group. It was envisioned that the diarylmethine stereocenter at C7′ in **6** could be formed by an Evans’ auxiliary-induced asymmetric conjugate addition of α,β-unsaturated acyl oxazolidinone **7** with 3,4,5-trimethoxyphenylmagnesium bromide (**8**). Both of these two building blocks could be conveniently prepared from commercially available 2,6-dimethoxyphenol [18–19].

### Synthesis of cyclization precursor **5** and (+)-aglacin B

**Scheme 2:**
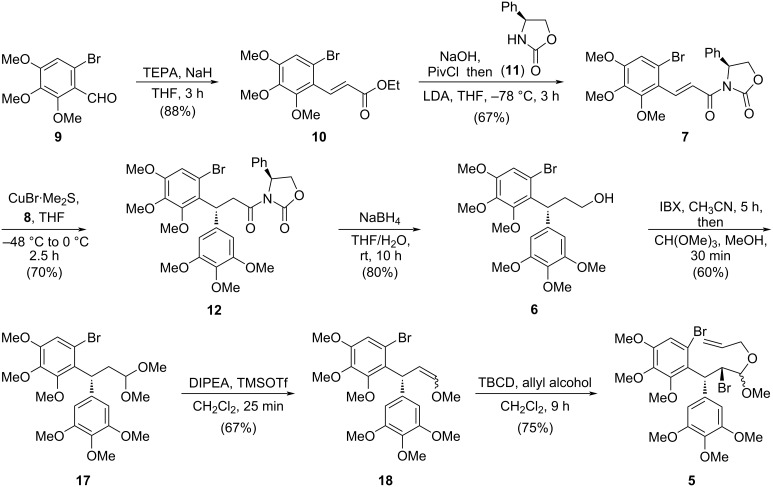
Synthesis of cyclization precursor **5**.

As shown in Scheme 2, the forward synthesis began with a triethyl phosphonoacetate-mediated Horner–Wadsworth–Emmons (HWE) reaction of *o*-bromobenzaldehyde **9** derived from 2,6-dimethoxyphenol (Supporting Information File 1). The generated ester **10** was then converted into the corresponding acyl chloride by saponification and subsequent reaction with pivaloyl chloride. The resulting acyl chloride was then trapped by (*S*)-4-phenyl-2-oxazolidinone (**11**) to produce the desired α,β-unsaturated amide **7**. Next, the asymmetric conjugate addition was carried out [20–21]. The in situ generated aryl–copper(I) species was obtained under the action of CuBr·Me_2_S with Grignard reagent **8**, and then added to a THF solution of the α,β-unsaturated acyl oxazolidinone **7** at −48 °C. This reaction demonstrated an excellent diastereocontrol for **12** (dr = 20:1), and could easily proceed on a scale of ten grams (Supporting Information File 1). For the reduction of the chiral auxiliary in **12**, NaBH_4_ in THF/H_2_O proved to be the optimal conditions, giving the primary alcohol **6** in 80% yield. Subsequently, oxidation of this alcohol by IBX followed by reaction with CH(OMe)_3_ afforded acetal **17**, which was then subjected to a CH_2_Cl_2_ solution of TMSOTf and iPr_2_NEt. A mixture of enol methyl ethers **18** were thus produced by an elimination reaction. Eventually, a site-selective bromination of the double bond over the electron-rich benzene rings with 2,4,4,6-tetrabromo-2,5-cyclohexadienone (TBCD) in CH_2_Cl_2_ followed by reaction with allyl alcohol, provided β-bromo acetal **5** in 30% overall yield starting from alcohol **6**.

**Scheme 3:**
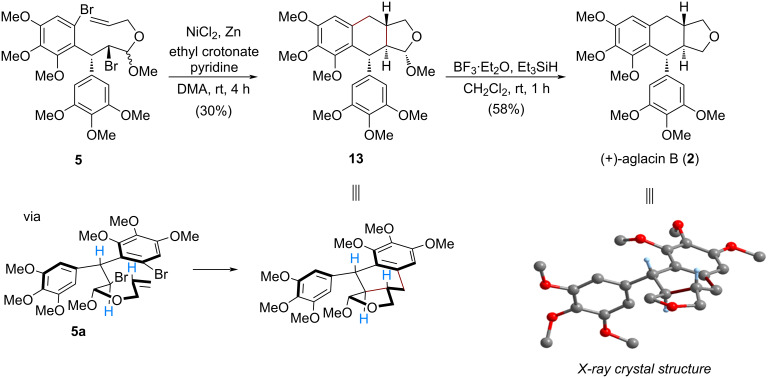
Synthesis of (+)-aglacin B (**2**).

With a successful preparation of the cyclization precursor **5**, the designed nickel-promoted reductive tandem cyclization was pursued (Scheme 3). By slightly modifying the reaction conditions of our previous studies [11–12], the expected bicyclization of **5** occurred smoothly, resulting in an efficient construction of the *trans*-tetrahydronaphtho[2,3-*c*]furan skeleton embedded in **13**, which could be separated from the other diastereomer [14] by flash column chromatography in 30% yield. The stereocontrolled formation of aryltetralin **13** could be attributed to an adoption of a *pseudo*-half-chair conformation **5a**. Finally, the final step towards the total synthesis of (+)-aglacin B (**2**) was achieved by treatment with BF_3_·Et_2_O as the Lewis acid and Et_3_SiH as the hydrogen source [22], affording this natural product in 58% isolated yield. NMR data of the synthetic sample were found to be in agreement with those of previous literature (Tables S1 and S2, Supporting Information File 1). Moreover, the newly synthesized (+)-aglacin B (**2**) formed single crystals, and a corresponding X-ray diffraction analysis (inset in Scheme 3, selected H atoms have been omitted for clarity, and Table S3, Supporting Information File 1) unambiguously confirmed its precise structure with three continuous chiral centers.

## Conclusion

In summary, the total synthesis of (+)-aglacin B, a typical aryltetralin natural product [23–24], was completed from 2,6-dimethoxyphenol. The key Ni-promoted reductive cyclization cascade of a β-bromo acetal with an allyl tether, smoothly established the tetrahydronaphtho[2,3-*c*]furan core of this molecule in a new fashion.

## Supporting Information

File 1 Experimental procedures, characterization data, and copies of ^1^H/^13^C NMR spectra.

## Data Availability

All data that supports the findings of this study is available in the published article and/or the supporting information of this article.
