# Supplementary material for: Ni-promoted reductive cyclization cascade enables a total synthesis of (+)-aglacin B
Source: Beilstein J Org Chem. 2025 Nov 18;21:2548–52. doi: 10.3762/bjoc.21.197 (PMC12642946; doi:10.3762/bjoc.21.197)
Supplement: File 1 — Experimental procedures, characterization data, and copies of 1H/13C NMR spectra. [file Beilstein_J_Org_Chem-21-2548-s001.pdf]

BEILSTEIN JOURNAL OF ORGANIC CHEMISTRY

Supporting Information

for

Ni-promoted reductive cyclization cascade enables a total synthesis of (+)-aglacin B

Si-Chen Yao, Jing-Si Cao, Jian Xiao, Ya-Wen Wang and Yu Peng

Beilstein J. Org. Chem. **2025,** *21,* 2548–2552. doi:10.3762/bjoc.21.197

Experimental procedures, characterization data, and copies of $^{1}H/^{13}C$ NMR spectra

License and Terms: This is a supporting information file under the terms of the Creative Commons Attribution License (https://creativecommons.org/licenses/by/4.0). Please note that the reuse, redistribution and reproduction in particular requires that the author(s) and source are credited and that individual graphics may be subject to special legal provisions.
The license is subject to the *Beilstein Journal of Organic Chemistry* terms and conditions: (https://www.beilstein-journals.org/bjoc/terms)

Table of contents

- General procedure......S2
- Preparation of *o*-bromobenzaldehyde **9**......S3
- Preparation of unsaturated amide **7**......S4-S6
- Asymmetric synthesis of amide **12**......S6
- Synthesis of alcohol **6**......S7
- Synthesis of acetal **17**......S8
- Synthesis of enol ether **18**......S9
- Preparation of cyclization precursor **5**......S10
- Synthesis of *trans*-THN[2,3-*c*]furan skeleton **13**...... S11
- Synthesis of (+)-aglacin B (**2**)......S12
- NMR data comparison of (+)-aglacin B (**2**, Tables S1 and S2)......S13-S14
- X-ray crystal data of (+)-aglacin B (**2**, Table S3)......S15
- ^{1}H NMR spectra of **16**......S16
- ^{1}H NMR and ^{13}C NMR spectra of **9**......S17-S18
- ^{1}H NMR and ^{13}C NMR spectra of **10**......S19-S20
- ^{1}H NMR and ^{13}C NMR spectra of **7**......S21-S22
- ^{1}H NMR and ^{13}C NMR spectra of **12**......S23-S24
- ^{1}H NMR spectra of **6**......S25
- ^{1}H NMR and ^{13}C NMR spectra of **17**......S26-S27
- ^{1}H NMR and ^{13}C NMR spectra of **18**......S28-S29
- ^{1}H NMR spectra of **5**......S30
- ^{1}H NMR and ^{13}C NMR spectra of **13**......S31-S32
- ^{1}H NMR and ^{13}C NMR spectra of (+)-aglacin B(**2**)......S33-S34

General procedure

For product purification by flash column chromatography, silica gel (200–300 mesh) from the Qingdao Hailang silicon material company in China, and petroleum ether (bp 60–90 °C) from Chengdu Lixinhe Chemical Co., Ltd. were used. All solvents were purified and dried by standard techniques and distilled prior to use. All experiments were conducted under an argon or nitrogen atmosphere in oven-dried or flame-dried glassware with magnetic stirring, unless otherwise specified. Organic extracts were dried over Na_2SO_4, unless otherwise noted. ^1H and ^{13}C NMR spectra were taken on a Bruker AM-400 with TMS as an internal standard and $CDCl_3$ as solvent unless otherwise noted. HRMS were determined on a Bruker Daltonics APEXII 47e FT-ICR spectrometer with ESI positive ion mode. The X-ray diffraction studies were carried out on a Bruker SMART Apex CCD area detector diffractometer equipped with graphite-monochromated Cu Kα radiation source. The specific optical rotation was obtained from Rudolph Research Analytical Autopol VI automatic polarimeter.

Preparation of *o*-bromobenzaldehyde 9

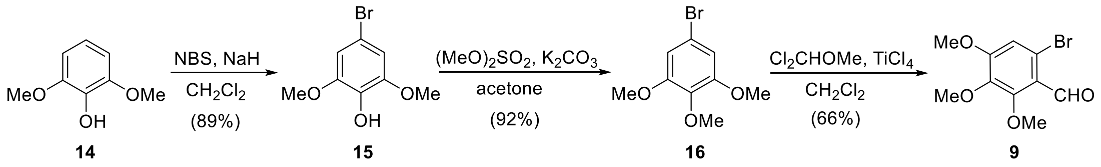

o-Bromobenzaldehyde **9** was prepared according to a general protocol described by Masse and co-workers[1]. In a 250 mL round-bottomed flask, 2,6-dimethoxyphenol (**14**, 10.0 g, 64.86 mmol) was dissolved in anhydrous CH_2Cl_2 (100 mL) and cooled to −45 °C. To this solution was added NaH (60%, 0.03 g, 0.65 mmol, 0.01 equiv) in MeOH (2 mL) and NBS (99%, 11.66 g, 64.86 mmol, 1.0 equiv) portionwise, and the mixture was stirred for 30 minutes at −45 °C. The resulting mixture was gradually warmed to room temperature and stirred further for 3 hours. The reaction was quenched by the addition of water (50 mL). The resulting mixture was then extracted with CH_2Cl_2 (2 × 50 mL), and the combined organic layers were washed with water (2 × 40 mL) and brine (40 mL) respectively, dried over Na_2SO_4, filtered and concentrated under reduced pressure. The crude product was purified by flash column chromatography (petroleum ether/EtOAc 8:1 → petroleum ether/EtOAc 4:1) on silica gel to afford **15** (13.45 g, 89% yield) as a white solid. R_f = 0.6 (petroleum ether/EtOAc 2:1).

A dry 250 mL two-necked, round-bottomed flask equipped with a reflux condenser was charged with the above bromobenzene **15** (13.0 g, 55.78 mmol), K_2CO_3 (15.42 g, 111.56 mmol, 2.0 equiv) and acetone (150 mL) under argon. Then $(MeO)_2SO_2$ (99%, 7.9 mL, 83.67 mmol, 1.5 equiv) was added to the above smoothly stirred system, the generated mixture was then heated to reflux for 12 h until TLC indicated the reaction is completed. The mixture was concentrated and extracted with EtOAc (50 mL), and the organic layers were washed with 1 M HCl (2 × 40 mL). The hydrochloric acid layers were extracted with EtOAc (2 × 50 mL), and the combined organic layers were washed with water (2 × 40 mL) and brine (40 mL) respectively, dried over Na_2SO_4, filtered and concentrated under reduced pressure. The crude product was purified by flash column chromatography (petroleum ether/EtOAc 30:1 → petroleum ether/EtOAc 10:1) on

---

[1] Massé, P.; Choppin, S.; Chiummiento, L.; Colobert, F., Hanquet, G. *J. Org. Chem.* **2021**, *86*, 3033–3040.

silica gel to afford **16** (12.68 g, 92% yield) as a white solid. R_f = 0.8 (petroleum ether/EtOAc 4:1). ^{1}H NMR (400 MHz, $CDCl_3$): δ = 6.71 (s, 2H), 3.84 (s, 6H), 3.81 (s, 3H) ppm.

In a 500 mL round-bottomed flask, $TiCl_4$ (99%, 13.5 mL, 122.38 mmol, 2.4 equiv), dichloromethyl methyl ether (97%, 9.2 mL, 101.98 mmol, 2.0 equiv) and anhydrous CH_2Cl_2 (100 mL) were added at 0 °C. After stirring for 20 minutes at 0 °C, a solution of 3,4,5-trimethoxybenzene **16** (12.60 g, 50.99 mmol) in DCM (100 mL) was added dropwise slowly and stirred for 20 minutes at 0 °C. The resulting mixture was gradually warmed to room temperature, and stirred for 5 hours. 1 M HCl (50 mL) was added at 0 °C and reacted at this temperature for 20 min. The resulting mixture was then extracted with CH_2Cl_2 (2 × 50 mL), and the combined organic layers were washed with water (2 × 40 mL) and brine (40 mL) respectively, dried over Na_2SO_4, filtered and concentrated under reduced pressure. The crude product was purified by flash column chromatography (petroleum ether/EtOAc 80:1 → petroleum ether/EtOAc 50:1) on silica gel to afford **9** (9.23 g, 66% yield) as a light yellow solid. R_f = 0.4 (petroleum ether/EtOAc 8:1). ^{1}H NMR (400 MHz, $CDCl_3$): δ = 10.24 (s, 1H), 6.97 (s, 1H), 3.96 (s, 3H), 3.93 (s, 3H), 3.87 (s, 3H) ppm; ^{13}C NMR (100 MHz, $CDCl_3$): δ = 189.3, 157.8, 156.9, 142.0, 121.6, 119.6, 113.4, 62.4, 61.0, 56.4 ppm.

Preparation of unsaturated amide 7

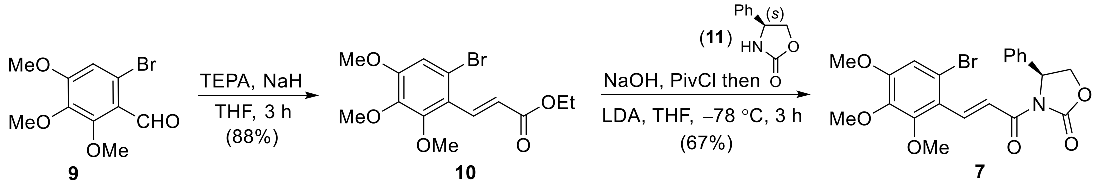

NaH (60%, 2.27 g, 56.85 mmol, 1.7 equiv) was added to anhydrous THF (60 mL) in a 200 mL round-bottomed flask, and the resulting slurry was cooled to 0 °C, followed by the addition of triethyl phosphonoacetate (98%, 11.3 mL, 76.85 mmol, 1.7 equiv) dropwise over a 6 min period. After stirring for 30 min at this temperature, a solution of *o*-bromobenzaldehyde **9** (9.23 g, 33.44 mmol) in THF (40 mL) was added dropwise. The resulting mixture was gradually warmed to room temperature, and stirred further for 2.5 h. The reaction was then quenched by the addition of water (20 mL). The

resultant mixture was extracted with EtOAc (2 × 50 mL), and the combined organic layers were washed with water (2 × 25 mL) and brine (20 mL), dried over Na_2SO_4, filtered and concentrated under reduced pressure. The crude product was purified by flash column chromatography (petroleum ether/EtOAc 40:1) on silica gel to afford **10** (8.66 g, 88% yield) as a white solid. R_f = 0.4 (petroleum ether/EtOAc 8:1). ^{1}H NMR (400 MHz, $CDCl_3$): δ = 7.85 (d, J = 16.0 Hz, 1H), 6.96 (s, 1H), 6.76 (d, J = 16.0 Hz, 1H), 4.27 (q, J = 7.2 Hz, 2H), 3.88 (s, 3H), 3.87 (s, 3H), 3.86 (s, 3H), 1.34 (t, J = 7.2 Hz, 3H) ppm; ^{13}C NMR (100 MHz, $CDCl_3$): δ = 167.6, 154.5, 154.2, 142.2, 139.7, 122.3, 121.4, 120.3, 112.5, 60.9, 60.6, 60.4, 56.2, 14.3 ppm; HRMS (ESI): calcd. for $C_{14}H_{17}BrO_5Na^+[M+Na]^+$: 367.0151, found: 367.0151.

To the above ester **10** (8.66 g, 29.42 mmol) dissolved in MeOH (80 mL) was added 3 M NaOH (30 mL). The resulting mixture was stirred for 3 h at 70 °C and then cooled to room temperature. The solvent was removed, and 6 N HCl (50 mL) was added dropwise. After stirring for 20 min, the mixture was filtered and the resulting white solid α,β-unsaturated acid (7.04 g) could be used directly for the next reaction without further purification.

Preparation of LDA: In a 100 mL round-bottomed flask, anhydrous THF (40 mL) was added at room temperature. After solvent was cooled to −78 °C, diisopropylamine (5.2 mL, 36.06 mmol, 1.3 equiv) was added and n-BuLi (2.5 M, 13.4 mL, 33.30 mmol, 1.2 equiv) was added dropwise slowly for 5 minutes. The mixture was reacted at this temperature for 30 min for further use.

In a 500 mL round-bottomed flask, (S)-4-phenyl-2-oxazolidinone (**11**, 99%, 5.49 g, 33.30 mmol, 1.2 equiv) was dissolved in anhydrous THF (70 mL) and the resulting solution was cooled to −78 °C. A solution of freshly prepared LDA (33.30 mmol, 1.2 equiv) was then added dropwise, with stirring for 1 h at the same temperature. The generated lithium amide was ready to use.

Meanwhile, triethylamine (4.7 mL, 33.30 mmol, 1.2 equiv) was added to a precooled (0 °C) THF (100 mL) solution of the above acid (7.0 g, 27.74 mmol) in a 250 mL round-bottomed flask, followed by the slow addition of pivaloyl chloride (4.1 mL, 33.30 mmol, 1.2 equiv). The mixture was stirred for 30 min at 0 °C, and the generated acyl chloride

was then added to the above lithium amide (−78 °C) slowly. The resulting mixture was gradually warmed to 0 °C, and stirred further for 3 h. The reaction was carefully quenched by the addition of saturated aqueous NH_4Cl solution (40 mL). The resultant mixture was extracted with EtOAc (2 × 60 mL), and the combined organic layers were washed with water (2 × 30 mL) and brine (20 mL) respectively, dried over Na_2SO_4, filtered and concentrated under reduced pressure. The crude product was purified by flash column chromatography (petroleum ether/EtOAc 15:1 → petroleum ether/EtOAc 10:1) on silica gel to afford **7** (11.6 g, 67% overall yield) as a white solid. R_f = 0.6 (petroleum ether/EtOAc 2:1). ^{1}H NMR (400 MHz, $CDCl_3$): δ = 8.27 (d, J = 16.0 Hz, 1H), 7.98 (d, J = 16.0 Hz, 1H), 7.41 – 7.31 (m, 5H), 6.95 (s, 1H), 5.55 (dd, J = 8.8, 4.0 Hz, 1H), 4.73 (t, J = 8.8 Hz, 1H), 4.30 (dd, J = 8.8, 4.0 Hz, 1H), 3.97 (s, 3H), 3.87 (s, 3H), 3.85 (s, 3H) ppm; ^{13}C NMR (100 MHz, $CDCl_3$): δ = 165.5, 155.0, 154.9, 153.7, 142.2, 141.5, 139.2, 129.1 (2C), 128.6, 126.0 (2C), 121.5, 121.2, 120.2, 112.5, 69.9, 60.8, 60.8, 57.9, 56.2 ppm; HRMS (ESI): calcd. for $C_{21}H_{20}BrNO_6Na^+[M+Na]^+$: 484.0366, found: 484.0364.

Asymmetric synthesis of amide 12

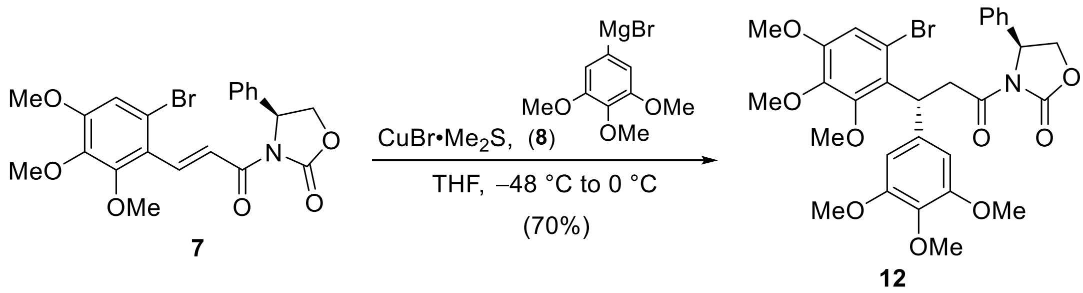

In a 250 mL three-necked round-bottomed flask, a 150 mL constant pressure drop funnel and reflux condenser were installed, Mg (9.63 g, 200.71 mmol, 8.0 equiv) and a grain of iodine were then added quickly to anhydrous THF (30 mL). After stirring for 3 minutes, under stirring a solution of the bromobenzene **16** (24.8 g, 100.35 mmol, 4.0 equiv) in anhydrous THF (100 mL) was added dropwise slowly over about 30 minutes in constant pressure drop funnel and gradually warmed to 66 °C, with stirring for 1 h at the same temperature. The generated Grignard reagent **8** was ready to

use.

Meanwhile, to a slurry of CuBr·SMe_2 (98%, 7.79 g, 37.63 mmol, 1.5 equiv) in THF (50 mL) at −48 °C were added dimethyl sulfide (50 mL) and freshly prepared 3,4,5-trimethoxyphenylmagnesium bromide **8**. The generated organocopper reagent was stirred further for 40 min at the same temperature, and a solution of unsaturated amide **7** (11.60 g, 25.09 mmol) in THF (100 mL) was then added dropwise. The resulting mixture was gradually warmed to 0 °C over a 30 min period, and stirred further for 2.5 h at 0 °C. The reaction was carefully quenched by the addition of saturated aqueous NH_4Cl solution (50 mL). The resulting mixture was extracted with EtOAc (2 × 40 mL), and the combined organic layers were washed with 10% aq $NH_3 \cdot H_2O$ (25 mL), water (2 × 25 mL) and brine (20 mL) respectively, dried over Na_2SO_4, filtered and concentrated under reduced pressure. The crude product was purified by flash column chromatography (petroleum ether/EtOAc 10:1 → 5:1) on silica gel to afford **12** (11.07 g, 70% yield) as a white solid. R_f = 0.4 (petroleum ether/EtOAc 2:1). $[\alpha]_D^{20}$ = +71.9 (c = 0.32, $CHCl_3$). ^1H NMR (400 MHz, $CDCl_3$): δ = 7.30 – 7.27 (m, 3H), 7.20 – 7.17 (m, 2H), 6.86 (s, 1H), 6.49 (s, 2H), 5.42 (dd, J = 8.4, 3.6 Hz, 1H), 5.18 (dd, J = 8.8, 6.0 Hz, 1H), 4.65 (t, J = 8.8 Hz, 1H), 4.29 – 4.23 (m, 2H), 3.82 (s, 3H), 3.78 (s, 3H), 3.77 (s,3H), 3.68 (s, 6H), 3.59 (s, 3H), 3.50 (dd, J = 17.2, 6.0 Hz, 1H) ppm; ^{13}C NMR (100 MHz, $CDCl_3$): δ = 171.3, 153.7, 153.4, 152.7, 152.6 (2C), 142.2, 138.7, 138.2, 136.3, 129.1, 129.0 (2C), 128.5, 125.8 (2C), 118.5, 111.7, 104.8 (2C), 69.9, 60.7, 60.7, 60.4, 57.6, 56.0, 55.9 (3C), 38.7 ppm; HRMS (ESI): calcd. for $C_{30}H_{32}BrNO_9Na^+$[M+Na]$^+$: 652.1152, found: 652.1161.

Synthesis of alcohol 6

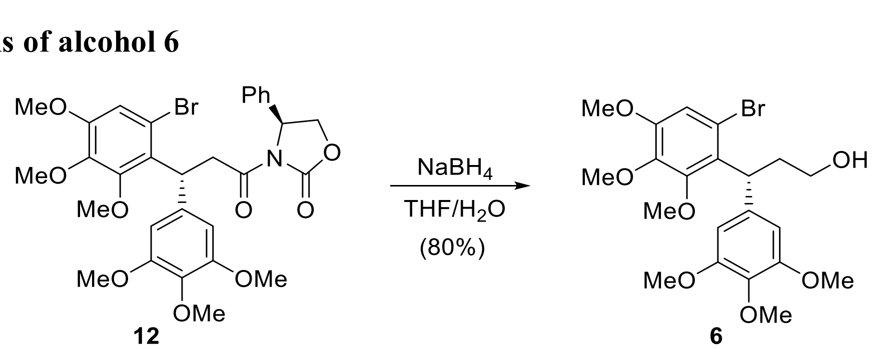

12 **6**

To a solution of **12** (11.07 g, 17.56 mmol) in THF (300 mL) was added water (30 mL)

followed by the addition of $NaBH_4$ (96%, 6.92 g, 175.56 mmol, 10.0 equiv) portionwise. The reaction temperature was kept at room temperature. 10 h later, THF was evaporated in vacuo. The resulting mixture was extracted with EtOAc (2 × 50 mL), and the combined organic layers were washed with water (2 × 25 mL) and brine (20 mL) respectively, dried over Na_2SO_4, filtered and concentrated under reduced pressure. The crude product was purified by flash column chromatography (petroleum ether/EtOAc 5:1) on silica gel to afford **6** (6.62 g, 80% yield) as a yellow oil. R_f = 0.7 (petroleum ether/EtOAc 1:1). $[\alpha]_D^{20}$ = +81.4 (c = 0.49, $CHCl_3$). ^1H NMR (400 MHz, $CDCl_3$) δ = 6.90 (s, 1H), 6.57 (s, 2H), 4.71 (s, 1H), 3.85 (s, 3H), 3.82 (s, 6H), 3.81 (s, 3H), 3.79 (s, 3H), 3.69 – 3.56 (m, 2H), 3.38 (s, 1H), 2.54 – 2.43 (m, 2H) ppm.

Synthesis of acetal 17

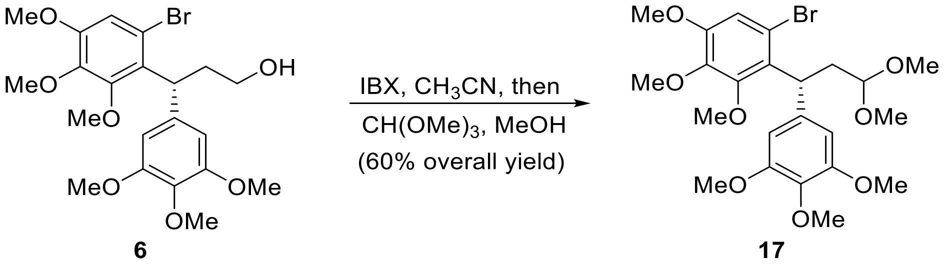

In a 250 mL round-bottomed flask, alcohol **6** (6.62 g, 14.05 mmol) was dissolved in anhydrous MeCN (100 mL) followed by the addition of IBX (95%, 12.43 g, 42.15 mmol, 3.0 equiv) portionwise at 0 °C. The resulting mixture was allowed to slowly warm to room temperature, and stirred further for 5 h. The reaction mixture was then filtered through a short plug of silica gel, and the filtrate was concentrated under reduced pressure to give the aldehyde, which could be used directly for the next reaction without further purification.

The above aldehyde (14.05 mmol) was dissolved in anhydrous MeOH (100 mL) followed by the addition of trimethyl orthoformate (6 mL) dropwise and (±)-camphorsulfonic acid (99%, 0.33 g, 1.41 mmol, 0.1 equiv). The reaction mixture was stirred for 35 min at 45 °C, cooled to room temperature, and then concentrated under reduced pressure. The crude product was purified by flash column chromatography (petroleum ether/EtOAc 4:1) on silica gel to afford **17** (4.35 g, 60% overall yield) as a

yellow oil. R_f = 0.6 (petroleum ether/EtOAc 2:1). $[\alpha]_D^{20}$ = +64.2 (c = 0.46, $CHCl_3$). ^1H NMR (400 MHz, $CDCl_3$): δ = 6.91 (s, 1H), 6.60 (s, 2H), 4.68 (t, J = 7.2 Hz, 1H), 4.20 (dd, J = 6.8, 4.4 Hz, 1H), 3.84 (s, 3H), 3.82 (s, 6H), 3.80 (s, 3H), 3.79 (s, 3H), 3.33 (s, 3H), 3.27 (s, 3H), 2.64 – 2..57 (m, 1H), 2.51 – 2..44 (m, 1H) ppm; ^{13}C NMR (100 MHz, $CDCl_3$): δ = 153.3, 152.8, 152.7, 152.6, 142.3, 139.4, 138.4, 136.4, 136.3, 129.3, 104.9 , 104.7, 103.4, 60.8, 60.5, 56.1 (2C), 56.0, 53.1, 52.4, 51.7, 37.2, 34.8 ppm; HRMS (ESI): calcd. for $C_{23}H_{31}BrO_8Na^+[M+Na]^+$: 537.1094, found: 537.1096.

Synthesis of enol ether 18

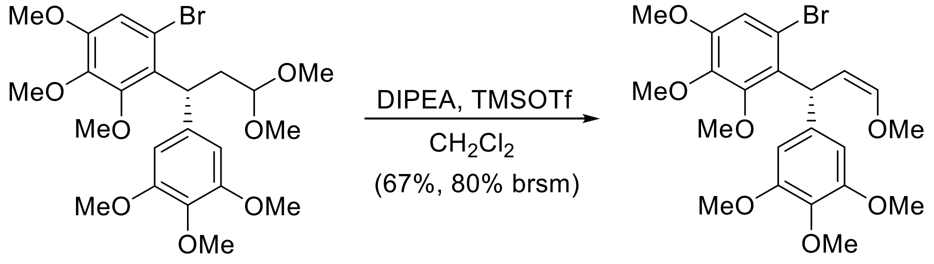

17 **18**

In a 100 mL round-bottomed flask, the acetal **17** (4.34 g, 8.42 mmol) and *N*-ethyl diisopropylamine (98%, 5.2 mL, 29.47 mmol, 3.5 equiv) were dissolved in CH_2Cl_2 (40 mL). After the resulting solution was cooled to 25 °C, TMSOTf (98%, 4.6 mL, 25.26 mmol, 3.0 equiv) was then added dropwise through a syringe with stirring. The pale yellow mixture was stirred for 25 min at this temperature. The reaction was quenched by the addition of a saturated aqueous $NaHCO_3$ solution (20 mL). The resultant mixture was extracted with CH_2Cl_2 (2 × 20 mL), and the combined organic layers were washed with water (2 × 10 mL) and brine (10 mL) respectively, dried over Na_2SO_4, filtered and concentrated under reduced pressure. The crude product was purified by flash column chromatography (petroleum ether/EtOAc 12/1) on silica gel to afford **18** (2.73 g, 67% yield, brsm 80%, *Z:E* = 10:1) as a yellow oil. R_f = 0.5 (petroleum ether/EtOAc 2:1). ^1H NMR (400 MHz, $CDCl_3$): δ = 6.90 (s, 1H), 6.47 (s, 2H), 6.11 (d, J = 6.0 Hz, 1H), 5.75 (d, J = 9.2 Hz, 1H), 5.17 (dd, J = 9.2, 6.0 Hz, 1H), 3.85 (s, 3H), 3.80 (s, 3H), 3.79 (s, 3H), 3.78 (s, 6H), 3.63 (s, 3H), 3.43 (s, 3H) ppm. ^{13}C NMR (100 MHz, $CDCl_3$): δ = 153.1, 152.9, 152.8 (2C), 152.5, 147.4, 142.4, 139.7,

136.9, 136.1, 130.8, 118.0, 106.6, 105.7, 104.4 (2C), 60.8, 60.5, 59.7, 56.2, 56.1 (2C) ppm; HRMS (ESI): calcd. for $C_{22}H_{27}BrO_7Na^+$[M+Na]$^+$: 505.0832, found: 505.0829.

Preparation of cyclization precursor 5

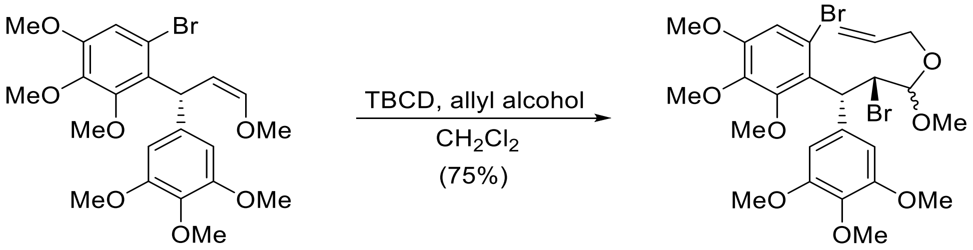

In a 200 mL round-bottomed flask, enol ether **18** (2.73 g, 5.64 mmol) was dissolved in anhydrous CH_2Cl_2 (50 mL) and cooled to 0 °C. To this solution was added TBCD (97%, 3.47 g, 8.46 mmol, 1.2 equiv) portionwise, and the mixture was stirred for 40 min at 0 °C. A solution of allyl alcohol (7.7 mL, 111.28 mmol, 20.0 equiv) in CH_2Cl_2 (5 mL) was then added dropwise, and the resulting mixture was gradually warmed to room temperature and stirred further for 9 h. The reaction was quenched with saturated aqueous $NaHCO_3$ (8 mL), Na_2SO_3 (8 mL) and stirred further for 30 min. The resulting mixture was extracted with CH_2Cl_2 (2 × 20 mL), and the combined organic layers were washed with water (2 × 10 mL) and brine (15 mL) respectively, dried over Na_2SO_4, filtered and concentrated under reduced pressure. The crude product was purified by flash column chromatography (petroleum ether/EtOAc 8:1) on silica gel to afford **5** (2.63 g, 75% yield, *dr* = 5 : 1) as a yellow oil. R_f = 0.5 (petroleum ether/EtOAc 2:1). ^{1}H NMR (400 MHz, $CDCl_3$): δ = 6.90 (s, 1H), 6.89 (s, 1H), 6.76 (s, 1H), 5.96 – 5.86 (m, 1H), 5.45 – 5.26 (m, 2H), 5.21 –5.16 (m, 1H), 5.05 – 5.01 (m, 1H), 4.12 (dd, J = 10.0, 6.0 Hz, 2H), 3.96 (s, 1H), 3.85 (s, 6H), 3.82 (s, 6H), 3.81 (s, 3H), 3.78 (s, 3H), 3.32 (s, 3H) ppm; HRMS (ESI): calcd. for $C_{25}H_{32}Br_2O_8Na^+$[M+Na]$^+$: 641.0356, found: 641.0359.

Synthesis of *trans*-THN[2,3-*c*]furan skeleton 13

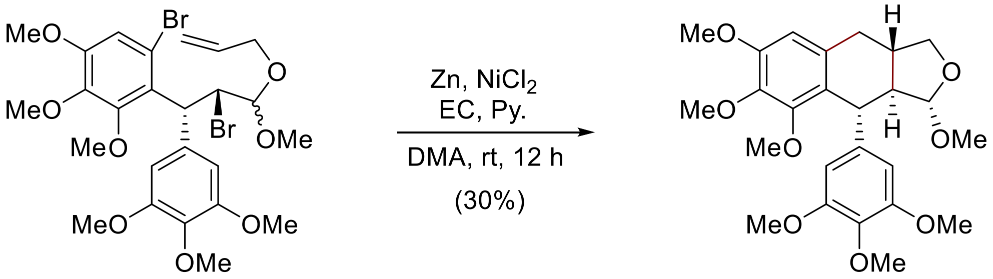

5 13

To a 100 mL round-bottomed flask were added $NiCl_2$ (0.56 g, 4.23 mmol, 1.0 equiv) and Zn (0.82 g, 12.67 mmol, 3.0 equiv). The flask was evacuated and then backfilled with argon. This process was repeated 4 times. Then pyridine (15 mL) and ethyl crotonate (1.6 mL, 12.67 mmol, 3.0 equiv) were added successively at room temperature. The temperature then rose to 55 °C, and stirring (350 r/min) was continued for 20 min. The resulting red-brown Ni(0) complex solution was cooled to room temperature, and a solution of β-bromo acetal **5** (2.63 g, 4.22 mmol) in DMA (30 mL) was added dropwise. After stirring for 4 h, the mixture was diluted and extracted with EtOAc (2 × 20 mL), and the combined organic layers were washed with water (4 × 15 mL) and brine (15 mL) respectively, dried over Na_2SO_4, filtered and concentrated. The crude product was purified by flash column chromatography (petroleum ether/EtOAc 5:1) on silica gel to afford **13** (0.58 g, 30% yield) as a yellow oil. $R_f = 0.3$ (petroleum ether/EtOAc 2:1). $[\alpha]_D^{20} = +32.8$ (c = 0.31, $CHCl_3$). ^1H NMR (400 MHz, $CDCl_3$): $\delta = 6.46$ (s, 1H), 6.29 (s, 2H), 4.95 (d, $J = 4.0$ Hz, 1H), 4.07 (t, $J = 4.8$ Hz, 1H), 3.99 (d, $J = 7.2$ Hz, 1H), 3.87 (s, 3H), 3.81 (s, 3H), 3.78 (s, 6H), 3.74 (s, 3H), 3.66 (dd, $J = 7.2$, 5.6 Hz, 1H), 3.21 (s, 3H), 3.14 (s, 3H), 2.84 – 2.81 (m, 2H), 2.16 – 2.11 (m, 1H), 1.92 (dt, $J = 11.6$, 4.0 Hz, 1H) ppm; ^{13}C NMR (100 MHz, $CDCl_3$): $\delta = 152.9$ (2C), 152.7, 152.3, 143.1, 140.8, 136.1, 132.8, 125.9, 109.9 (2C), 107.4, 104.2, 70.9, 60.9, 60.4, 59.4, 57.7, 56.1, 56.1 (2C), 55.8, 46.3, 41.9, 32.9 ppm; HRMS (ESI): calcd. for $C_{25}H_{32}O_8Na^+[M+Na]^+$: 483.1989, found: 483.2009.

Synthesis of (+)-aglacin B (2)

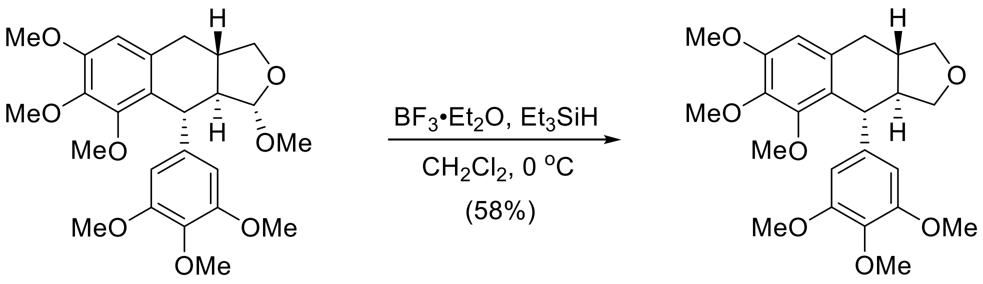

In a 50 mL round-bottomed flask, containing a solution of **13** (0.58 g, 1.27 mmol) in anhydrous CH_2Cl_2 (20 mL) was added dropwise at 0 °C Et_3SiH (99%, 4.1 mL, 25.4 mmol, 16.0 equiv) and $BF_3 \cdot Et_2O$ (1.3 mL, 10.16 mmol, 8.0 equiv). The reaction mixture was slowly warmed to room temperature over 1 hour and the reaction was quenched with saturated aqueous $NaHCO_3$ (15 mL). The resulting mixture was extracted with CH_2Cl_2 (2 × 20 mL), and the combined organic layers were washed with water (2 × 15 mL) and brine (15 mL) respectively, dried over Na_2SO_4, filtered and concentrated under reduced pressure. The crude product was purified by flash column chromatography (petroleum ether/EtOAc 4:1) on silica gel to afford (+)-aglacin B (**2**) (0.31 g, 58% yield) as a yellow solid. R_f = 0.3 (petroleum ether/EtOAc 1:1). $[\alpha]_D^{20}$ = +25.6 (*c* = 0.21, $CHCl_3$). ^1H NMR (400 MHz, $CDCl_3$): δ = 6.48 (s, 1H), 6.27 (s, 2H), 4.16 (t, *J* = 7.2 Hz, 1H), 3.91 (t, *J* = 7.6 Hz, 1H), 3.87 (s, 3H), 3.83 (d, *J* = 9.2 Hz, 1H), 3.81 (s, 3H), 3.78 (s, 6H), 3.74 (s, 3H), 3.60 (dd, *J* = 10.4, 8.0 Hz, 1H), 3.49 (dd, *J* = 10.0, 8.0 Hz, 1H), 3.15 (s, 3H), 2.92 (dd, *J* = 15.2, 4.0 Hz, 1H), 2.74 (dd, *J* = 15.6, 11.6 Hz, 1H), 2.15 – 2.07 (m, 1H), 2.06 – 1.96 (m, 1H), ppm; ^{13}C NMR (100 MHz, $CDCl_3$): δ = 153.1 (2C), 152.6, 152.3, 144.1, 140.8, 136.1 (2C), 133.0, 125.5, 107.4, 103.7, 72.7, 72.5, 60.9, 60.4, 59.4, 56.2 (2C), 55.8, 52.8, 46.9, 41.6, 33.4 ppm; HRMS (ESI): calcd. for $C_{24}H_{30}O_7Na^+[M+Na]^+$: 453.1884, found: 453.1900.

Table S1 ^{1}H NMR Comparison of synthetic (+)-aglacin B

^{1}H NMR [δ H (ppm), J (Hz)]			
**Natural** Aglacin B[2] ($CDCl_3$, 500 MHz)	**Gao's synthetic** Aglacin B[3] ($CDCl_3$, 500 MHz)	**Our synthetic** Aglacin B ($CDCl_3$, 400 MHz)	**Deviation** (natural–synthetic) Δδ (ppm)
6.49 *s*	6.48 *s*	6.48 (*s*, 1H)	0.01
6.27 *s*	6.27 *s*	6.27 (*s*, 2H)	0
4.16 br *t* (7.6)	4.17 *t* (7.5)	4.16 (*t*, *J* = 7.2 Hz, 1H)	0
3.91 br *t* (7.6)	3.92 *t* (7.4)	3.91 (*t*, *J* = 7.6 Hz, 1H)	0
3.86 *s*	3.87 *s*	3.87 (*s*, 3H)	-0.01
3.82 *d* (7.7)	3.84 *d* (10.7)	3.83 (*d*, *J* = 9.2 Hz, 1H)	-0.01
3.81 *s*	3.81 *s*	3.81 (*s*, 3H)	0
3.77 *s*	3.78 *s*	3.78 (*s*, 6H)	-0.01
3.73 *s*	3.74 *s*	3.74 (*s*, 3H)	-0.01
3.60 *dd* (10.1, 7.6)	3.60 *dd* (10.2, 7.7)	3.60 (*dd*, *J* = 10.4, 8.0 Hz, 1H)	0
3.49 *dd* (10.1, 7.9)	3.50 *dd* (10.2, 7.8)	3.49 (*dd*, *J* = 10.0, 8.0 Hz, 1H)	0
3.15 *s*	3.15 *s*	3.15 (*s*, 3H)	0
2.91 *dd* (15.5, 4.1)	2.92 *dd* (15.4, 4.1)	2.92 (*dd*, *J* = 15.2, 4.0 Hz, 1H)	-0.01
2.73 *dd* (15.1, 11.7)	2.74 *dd* (15.1, 11.9)	2.74 (*dd*, *J* = 15.6, 11.6 Hz, 1H)	-0.01
2.15-2.07 *m*	2.15-2.07 *m*	2.15-2.07 *m*	0
2.06-1.96 *m*	2.06-1.98 *m*	2.06-1.96 *m*	0

[2] Wang, B. G.; Ebel, R.; Nugroho, B. W.; Prijono, D.; Frank, W.; Steube, K. G.; Hao, X. J.; Proksch, P. *J. Nat. Prod.* **2001**, *64,* 1521-1526.
[3] Xu, M.; Hou, M.; He, H.; Gao, S. *Angew. Chem., Int. Ed.* **2021**, *60*, 16655-16660.

Table S2 ^{13}C NMR Comparison of synthetic (+)-aglacin B

^{13}C NMR [δ C (ppm)]			
**Natural** Aglacin B[2] ($CDCl_3$, 125 MHz)	**Gao's synthetic** Aglacin B[3] ($CDCl_3$, 125 MHz)	**Our synthetic** Aglacin B ($CDCl_3$, 100 MHz)	**Deviation** (natural–synthetic) Δδ (ppm)
153.1 (2C)	153.2 (2C)	153.1 (2C)	0
152.6	152.7	152.6	0
152.3	152.3	152.3	0
144.1	144.2	144.1	0
140.8	140.9	140.8	0
136.1	136.2	136.1	0
133.0	133.1	133.0	0
125.5	125.6	125.5	0
107.5	107.5	107.4	0.1
103.8 (2C)	103.8 (2C)	103.7 (2C)	0.1
72.7	72.8	72.7	0
72.6	72.6	72.5	0.1
60.9	61.0	60.9	0
60.4	60.5	60.4	0
59.4	59.5	59.4	0
56.2 (2C)	56.3 (2C)	56.2 (2C)	0
55.8	55.9	55.8	0
52.8	52.9	52.8	0
46.9	46.9	46.9	0
41.7	41.7	41.6	0.1
33.5	33.5	33.4	0.1

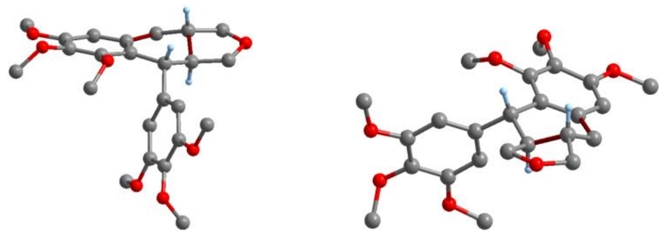

Table S3: X-ray crystal data of (+)-aglacin B

Empirical formula	$C_{24}H_{30}O_7$
Formula weight	430.48
Temperature (K)	296.99(10)
Crystal system	orthorhombic
Space group	$P2_12_12_1$
a (Å)	6.81047(8)
b (Å)	11.61724(17)
c (Å)	28.1265(3)
α (°)	90.00
β (°)	90.00
γ (°)	90.00
V (Å^3)	2225.34(5)
Z	4
Density (calculated) (g/cm^3)	1.285
μ (mm^{-1})	0.774
F (000)	920.0
Crystal size (mm^3)	0.14 × 0.13 × 0.12
Radiation	Cu Kα (λ = 1.54184)
2*θ* range for data collection (°)	6.284 to 155.236
Index ranges	$-8 \leq h \leq 4$<br>$-14 \leq k \leq 14$<br>$-32 \leq l \leq 35$
Reflections collected	8566
Independent reflections	4104 [R_{int} = 0.0185, R_{sigma} = 0.0223]
Data/restraints/parameters	4104/0/287
Goodness-of-fit on F^2	1.062
Final R indexes [I >= 2σ (*I*)]	R_1 = 0.0332, wR_2 = 0.0937
Final R indexes [all data]	R_1 = 0.0345, wR_2 = 0.0948
Largest diff. peak and hole [e Å^{-3}]	0.14/–0.13
Flack parameter	-0.04(7)

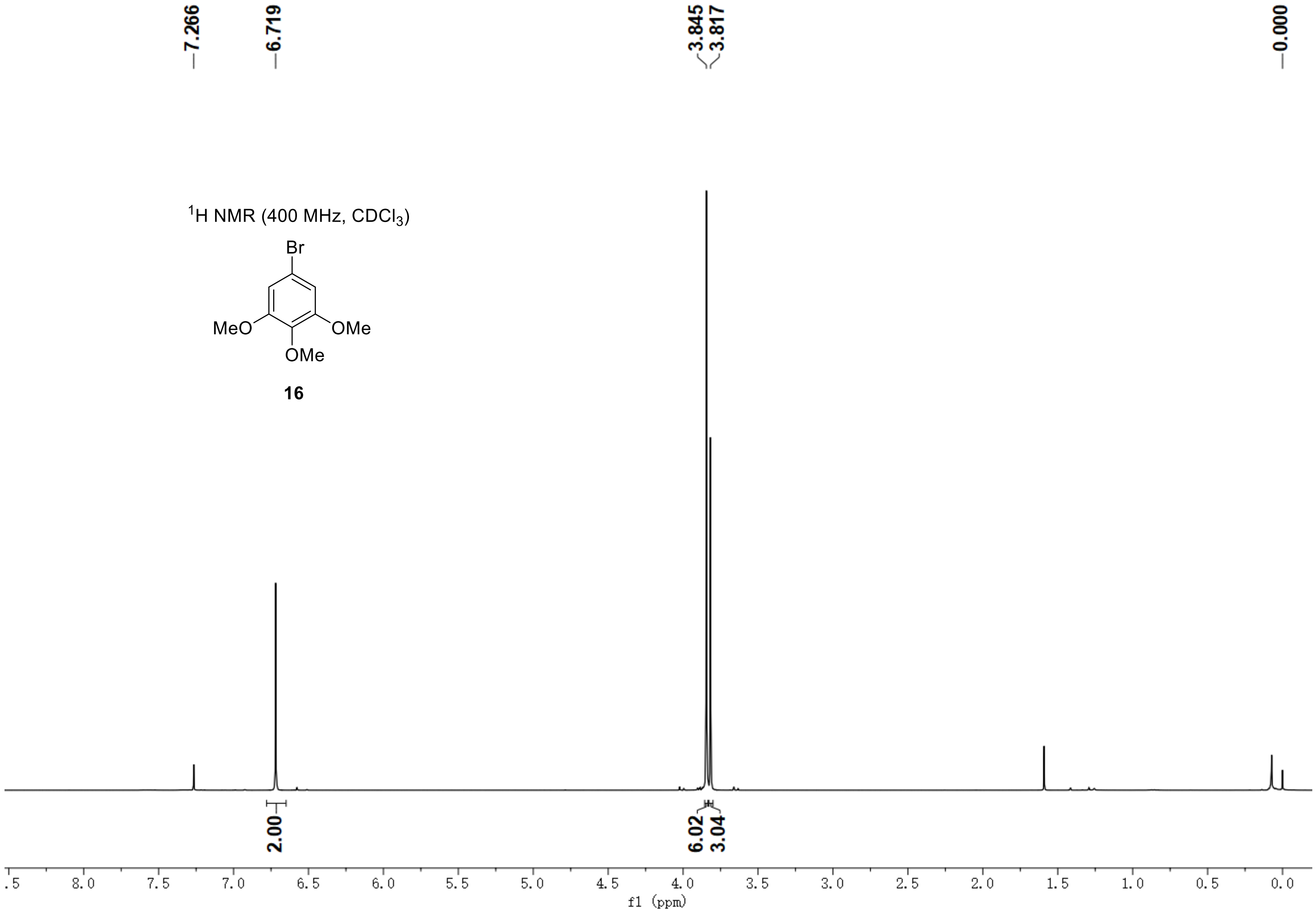
7.266
6.719
3.845
3.817
0.000
1H NMR (400 MHz, CDCl3)
Br
MeO
OMe
OMe
16
2.00
6.02
3.04
.5 8.0 7.5 7.0 6.5 6.0 5.5 5.0 4.5 4.0 3.5 3.0 2.5 2.0 1.5 1.0 0.5 0.0
f1 (ppm)

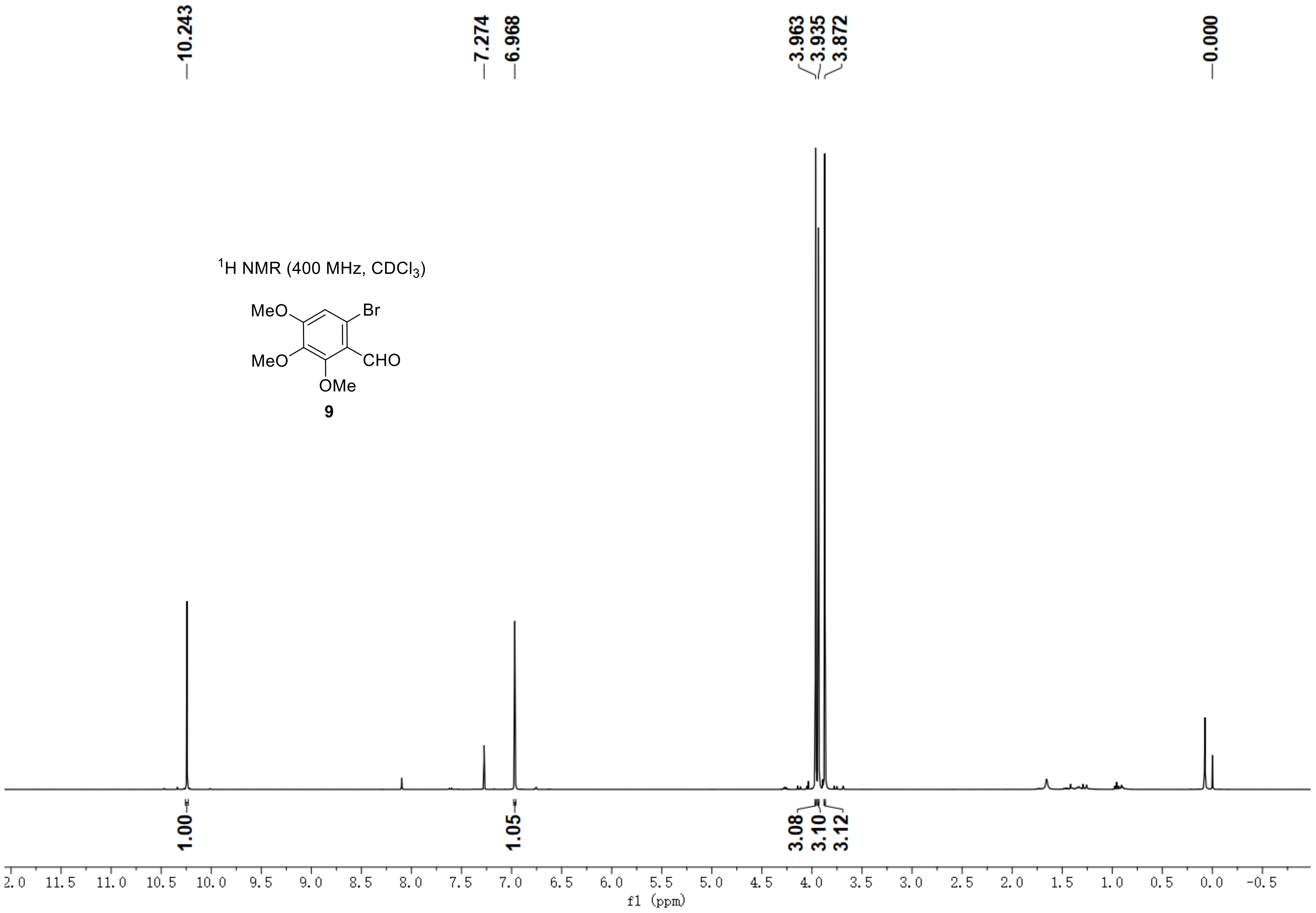

10.243
7.274
6.968
3.963
3.935
3.872
0.000
1H NMR (400 MHz, CDCl3)
MeO
Br
MeO
CHO
OMe
9
1.00
1.05
3.08
3.10
3.12
f1 (ppm)

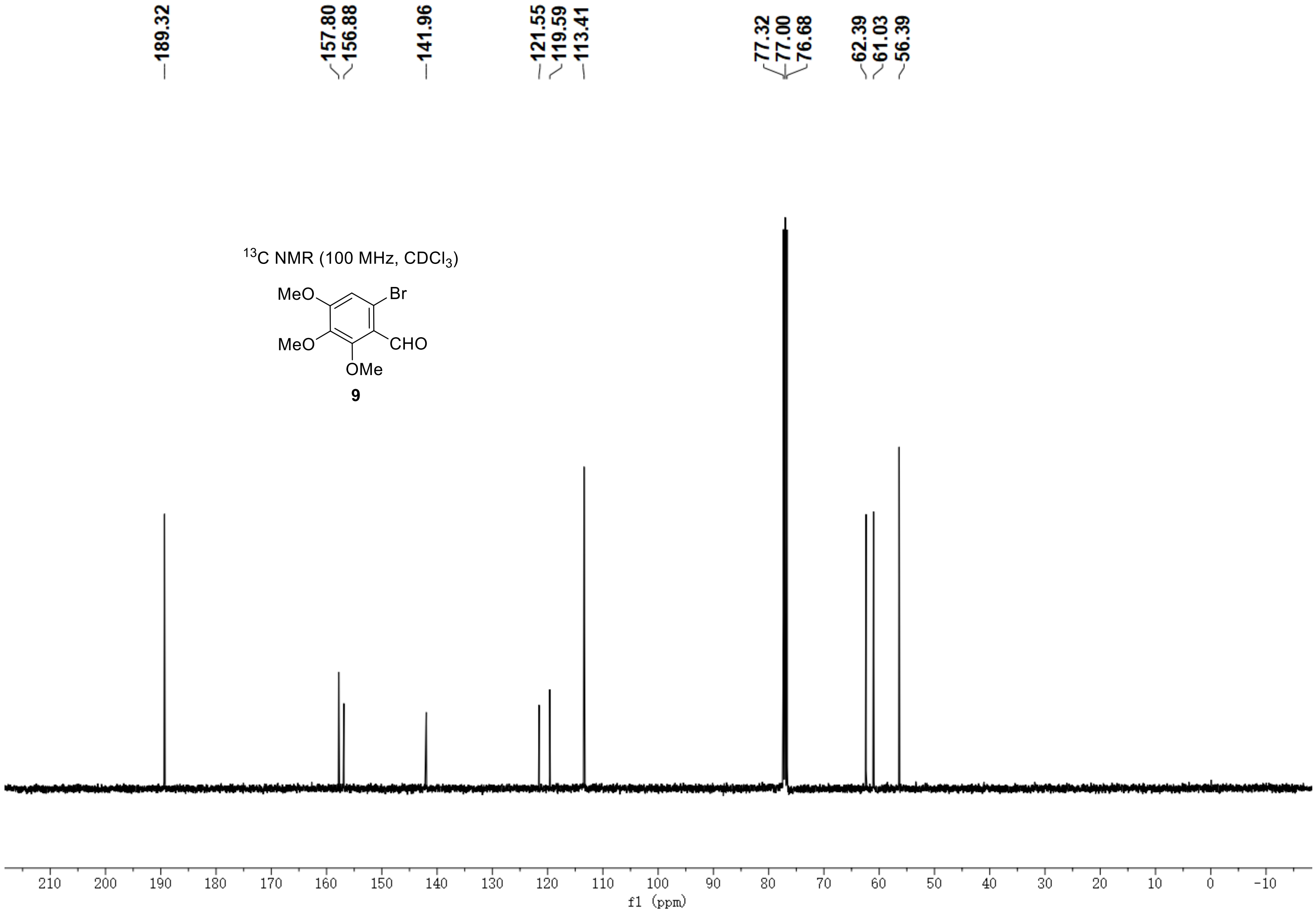
189.32
157.80
156.88
141.96
121.55
119.59
113.41
77.32
77.00
76.68
62.39
61.03
56.39
^{13}C NMR (100 MHz, $CDCl_3$)
MeO
Br
MeO
CHO
OMe
9
210 200 190 180 170 160 150 140 130 120 110 100 90 80 70 60 50 40 30 20 10 0 -10
f1 (ppm)

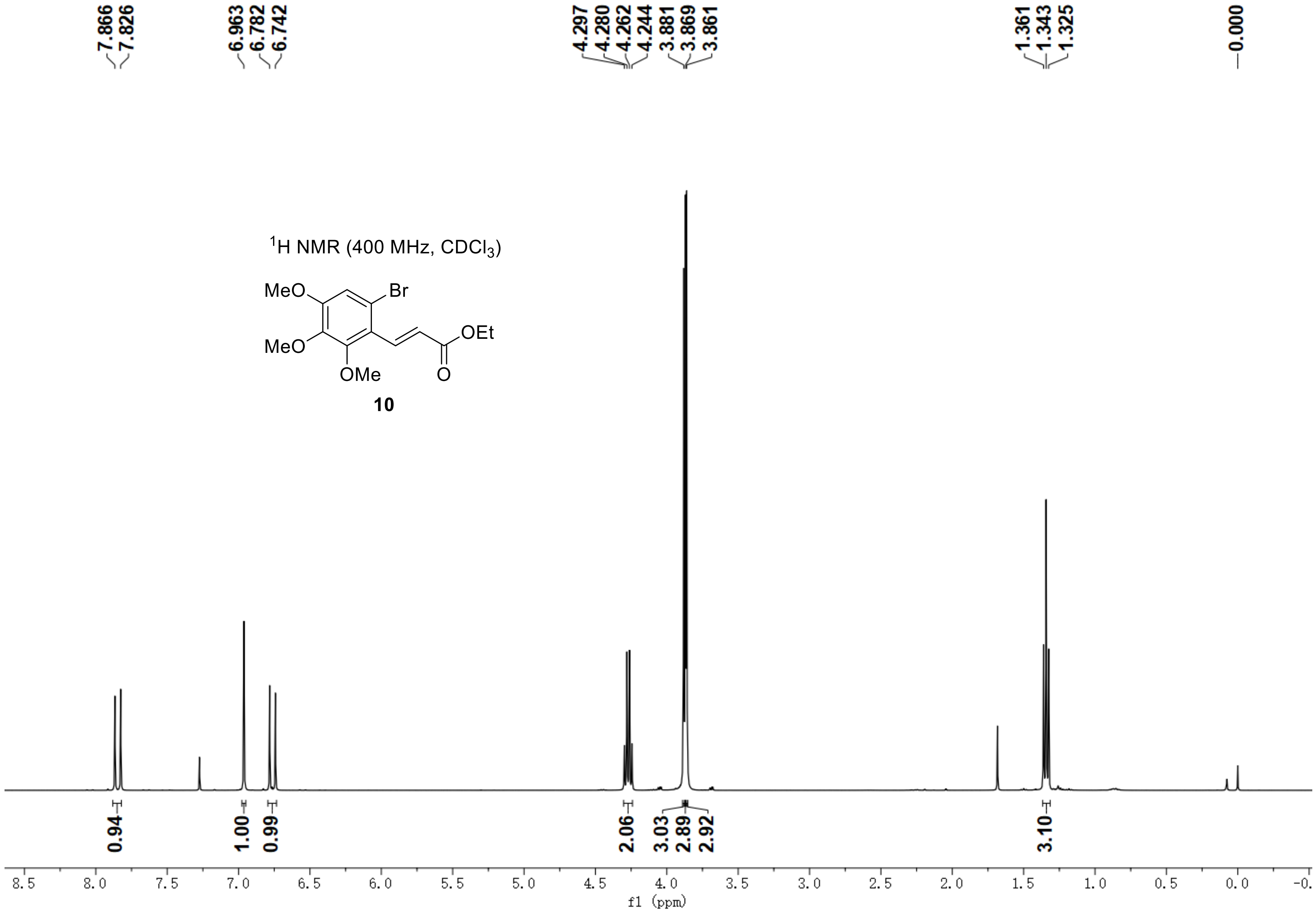
7.866
7.826
6.963
6.782
6.742
4.297
4.280
4.262
4.244
3.881
3.869
3.861
1.361
1.343
1.325
0.000
1H NMR (400 MHz, CDCl3)
MeO
Br
OEt
MeO
OMe
O
10
0.94
1.00
0.99
2.06
3.03
2.89
2.92
3.10
8.5
8.0
7.5
7.0
6.5
6.0
5.5
5.0
4.5
4.0
3.5
3.0
2.5
2.0
1.5
1.0
0.5
0.0
f1 (ppm)

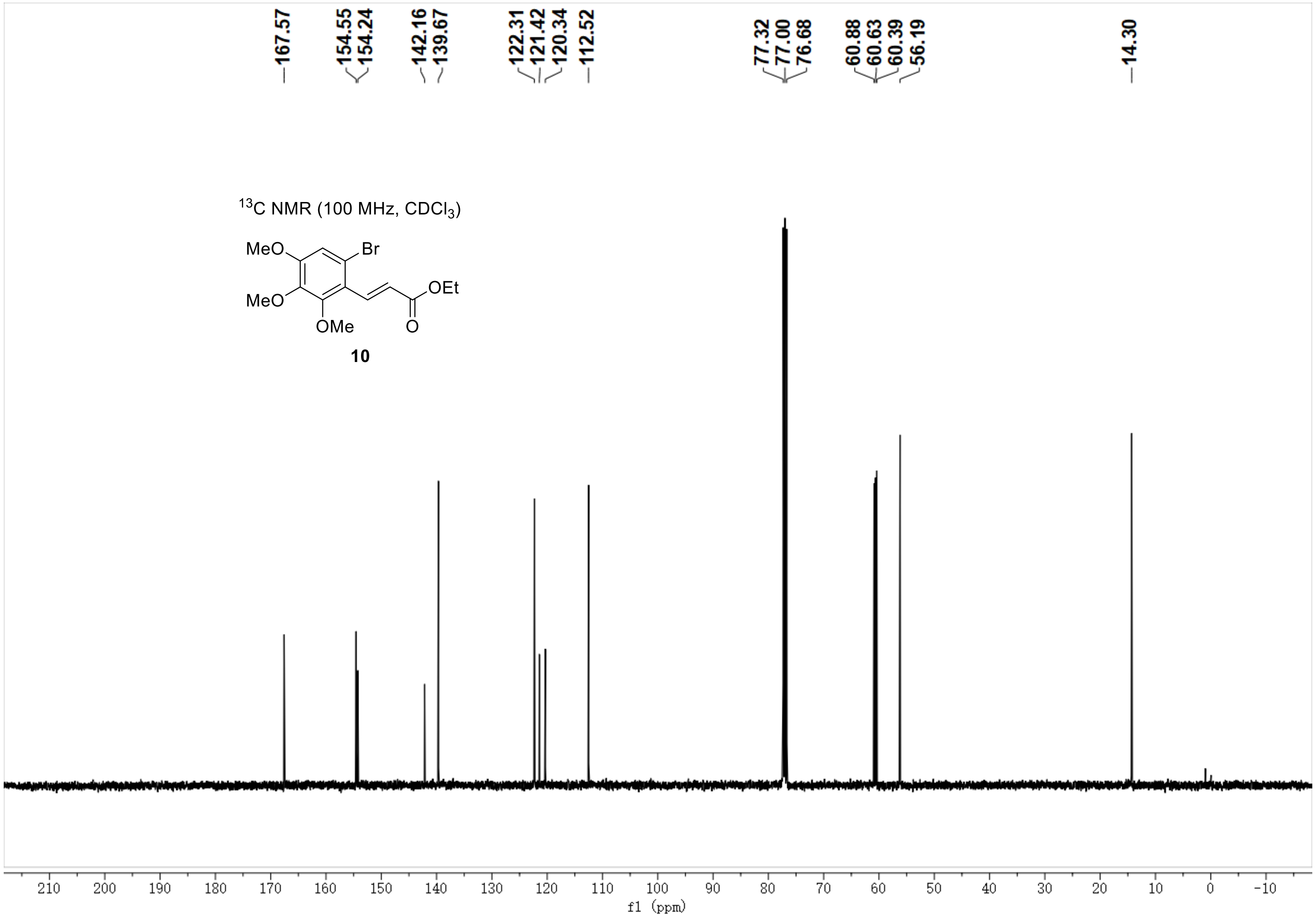
167.57
154.55
154.24
142.16
139.67
122.31
121.42
120.34
112.52
77.32
77.00
76.68
60.88
60.63
60.39
56.19
14.30
^{13}C NMR (100 MHz, CDCl$_3$)
MeO
Br
OEt
MeO
OMe
O
10
210 200 190 180 170 160 150 140 130 120 110 100 90 80 70 60 50 40 30 20 10 0 -10
f1 (ppm)

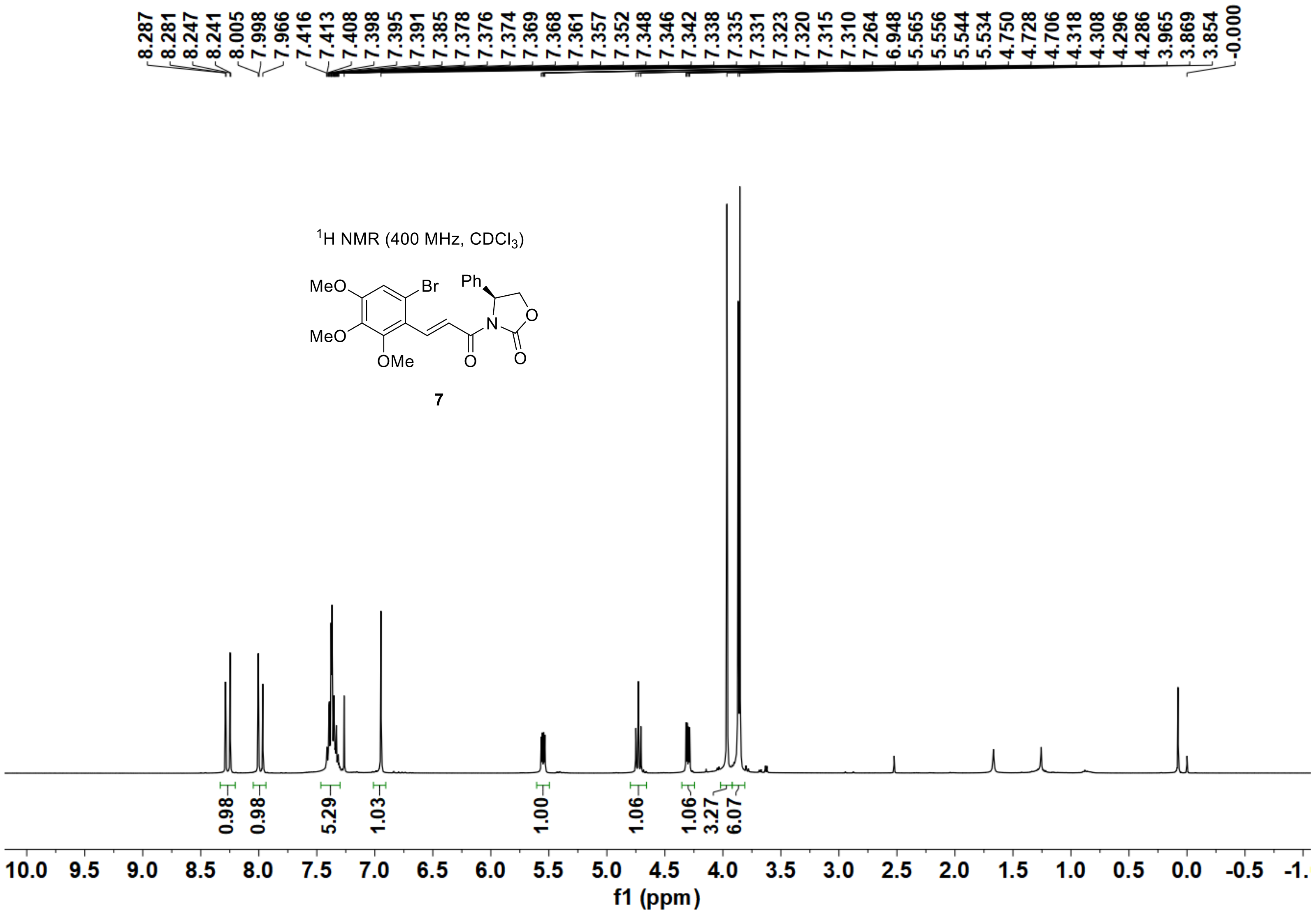

8.287
8.281
8.247
8.241
8.005
7.998
7.966
7.416
7.413
7.408
7.398
7.395
7.391
7.385
7.378
7.376
7.374
7.369
7.368
7.361
7.357
7.352
7.348
7.346
7.342
7.338
7.335
7.331
7.323
7.320
7.315
7.310
7.264
6.948
5.565
5.556
5.544
5.534
4.750
4.728
4.706
4.318
4.308
4.296
4.286
3.965
3.869
3.854
-0.000
^{1}H NMR (400 MHz, $CDCl_3$)
MeO
MeO
OMe
Br
Ph
N
O
O
O
0.98
0.98
5.29
1.03
1.00
1.06
1.06
3.27
6.07
10.0 9.5 9.0 8.5 8.0 7.5 7.0 6.5 6.0 5.5 5.0 4.5 4.0 3.5 3.0 2.5 2.0 1.5 1.0 0.5 0.0 -0.5 -1.
f1 (ppm)

7

^{13}C NMR (100 MHz, $CDCl_3$)

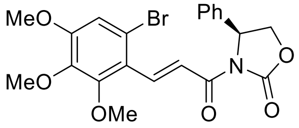

7

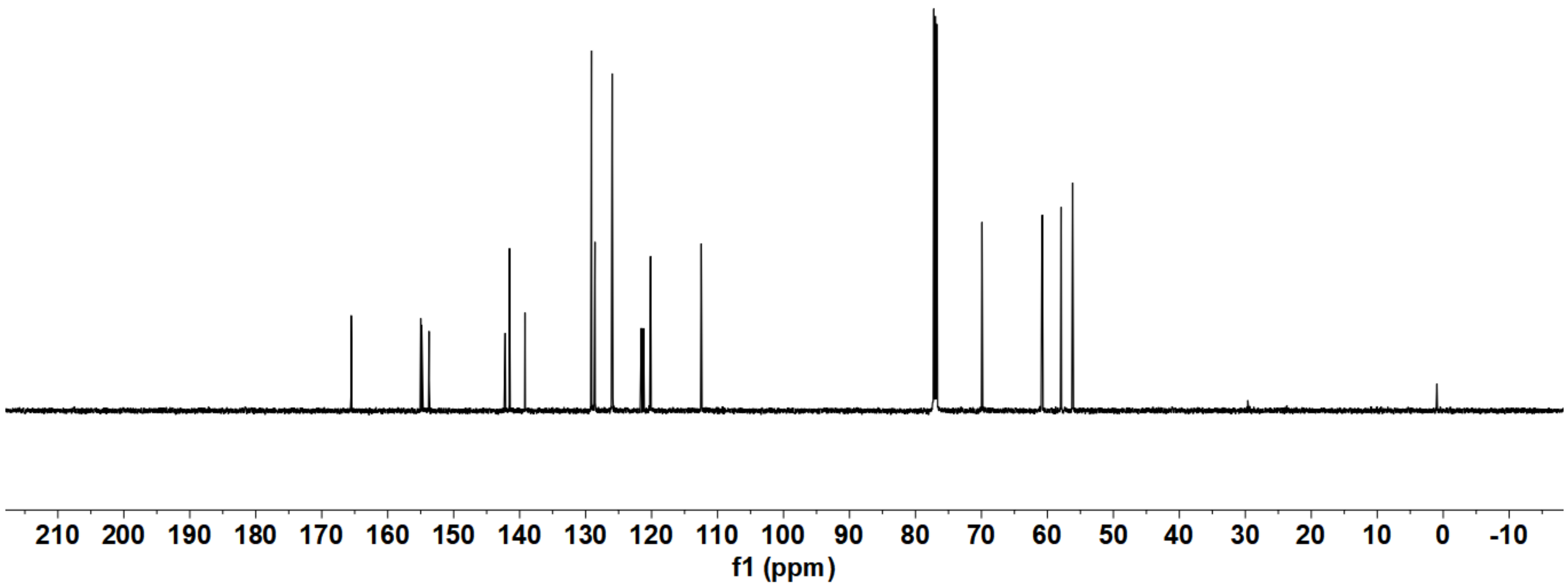

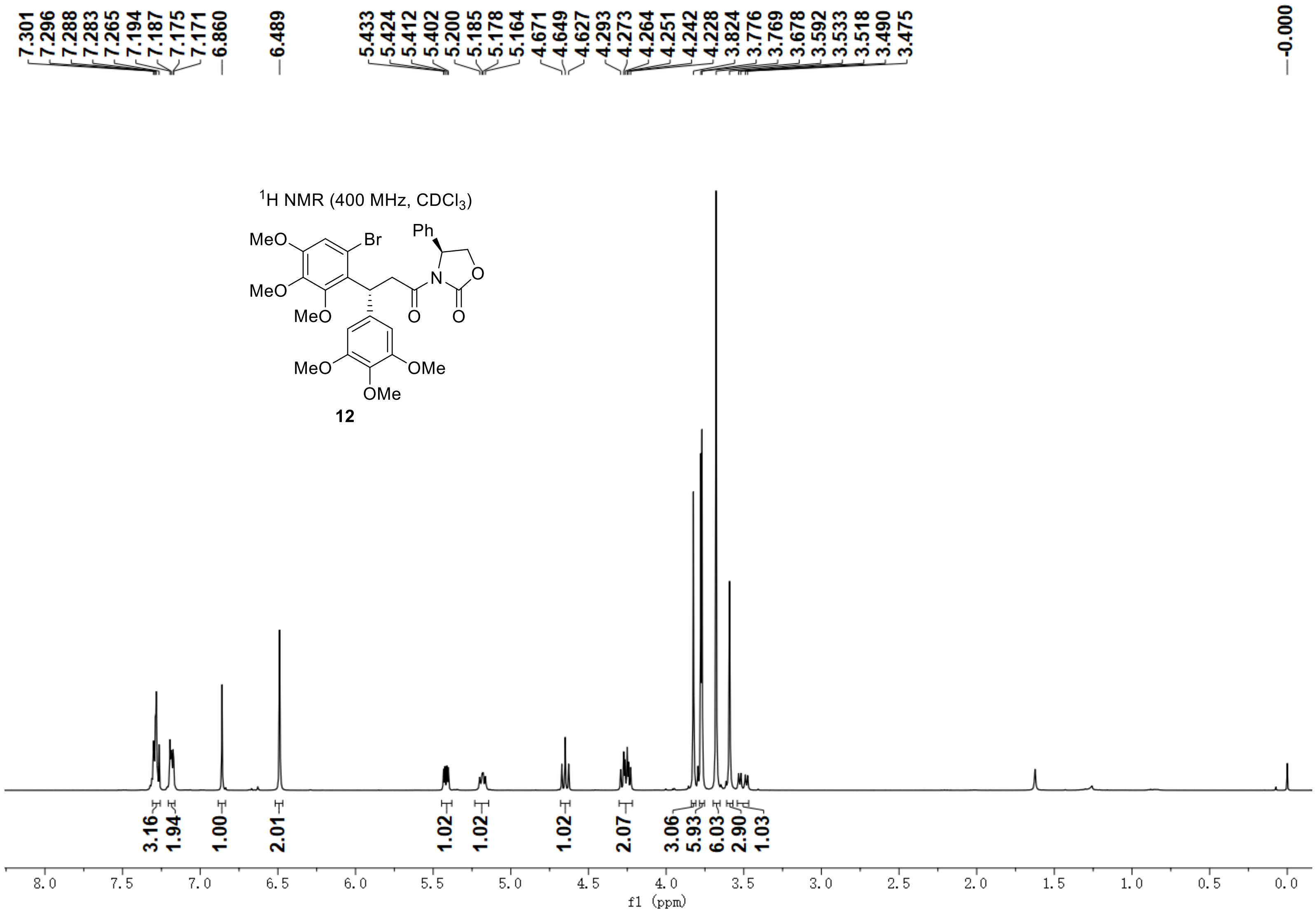
7.301
7.296
7.288
7.283
7.265
7.194
7.187
7.175
7.171
6.860
6.489
5.433
5.424
5.412
5.402
5.200
5.185
5.178
5.164
4.671
4.649
4.627
4.293
4.273
4.264
4.251
4.242
4.228
3.824
3.776
3.769
3.678
3.592
3.533
3.518
3.490
3.475
-0.000
^{1}H NMR (400 MHz, $CDCl_3$)
MeO
MeO
MeO
Br
Ph
N
O
O
O
MeO
OMe
OMe
12
3.16
1.94
1.00
2.01
1.02
1.02
1.02
2.07
3.06
5.93
6.03
2.90
1.03
8.0
7.5
7.0
6.5
6.0
5.5
5.0
4.5
4.0
3.5
3.0
2.5
2.0
1.5
1.0
0.5
0.0
f1 (ppm)

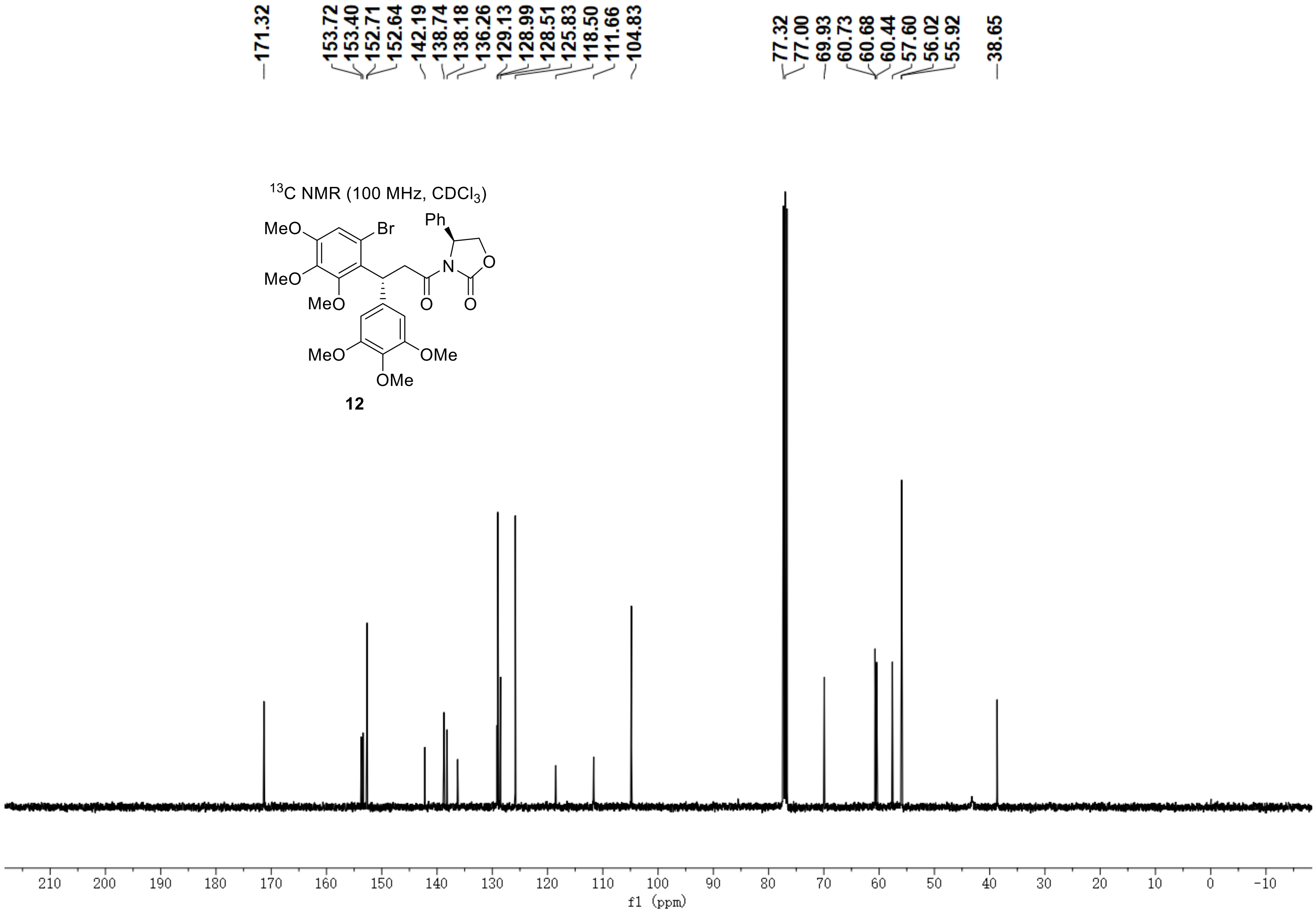
171.32
153.72
153.40
152.71
152.64
142.19
138.74
138.18
136.26
129.13
128.99
128.51
125.83
118.50
111.66
104.83
77.32
77.00
69.93
60.73
60.68
60.44
57.60
56.02
55.92
38.65
13C NMR (100 MHz, CDCl3)
MeO
MeO
MeO
Br
Ph
N
O
O
O
MeO
OMe
OMe
12
210 200 190 180 170 160 150 140 130 120 110 100 90 80 70 60 50 40 30 20 10 0 -10
f1 (ppm)

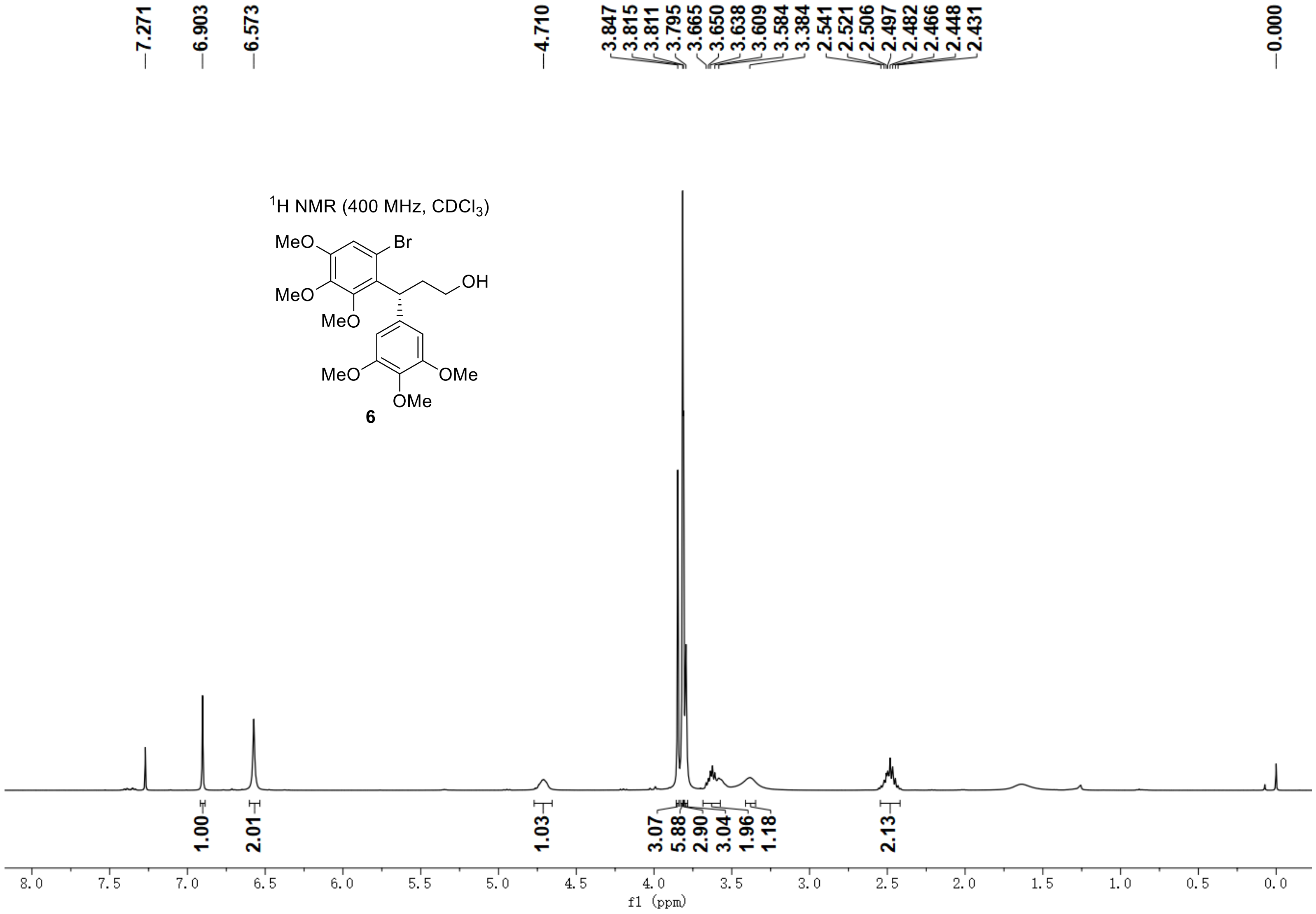
7.271
6.903
6.573
4.710
3.847
3.815
3.811
3.795
3.665
3.650
3.638
3.609
3.584
3.384
2.541
2.521
2.506
2.497
2.482
2.466
2.448
2.431
0.000
1H NMR (400 MHz, CDCl3)
MeO
Br
OH
MeO
MeO
MeO
OMe
OMe
6
1.00
2.01
1.03
3.07
5.88
2.90
3.04
1.96
1.18
2.13
8.0 7.5 7.0 6.5 6.0 5.5 5.0 4.5 4.0 3.5 3.0 2.5 2.0 1.5 1.0 0.5 0.0
f1 (ppm)

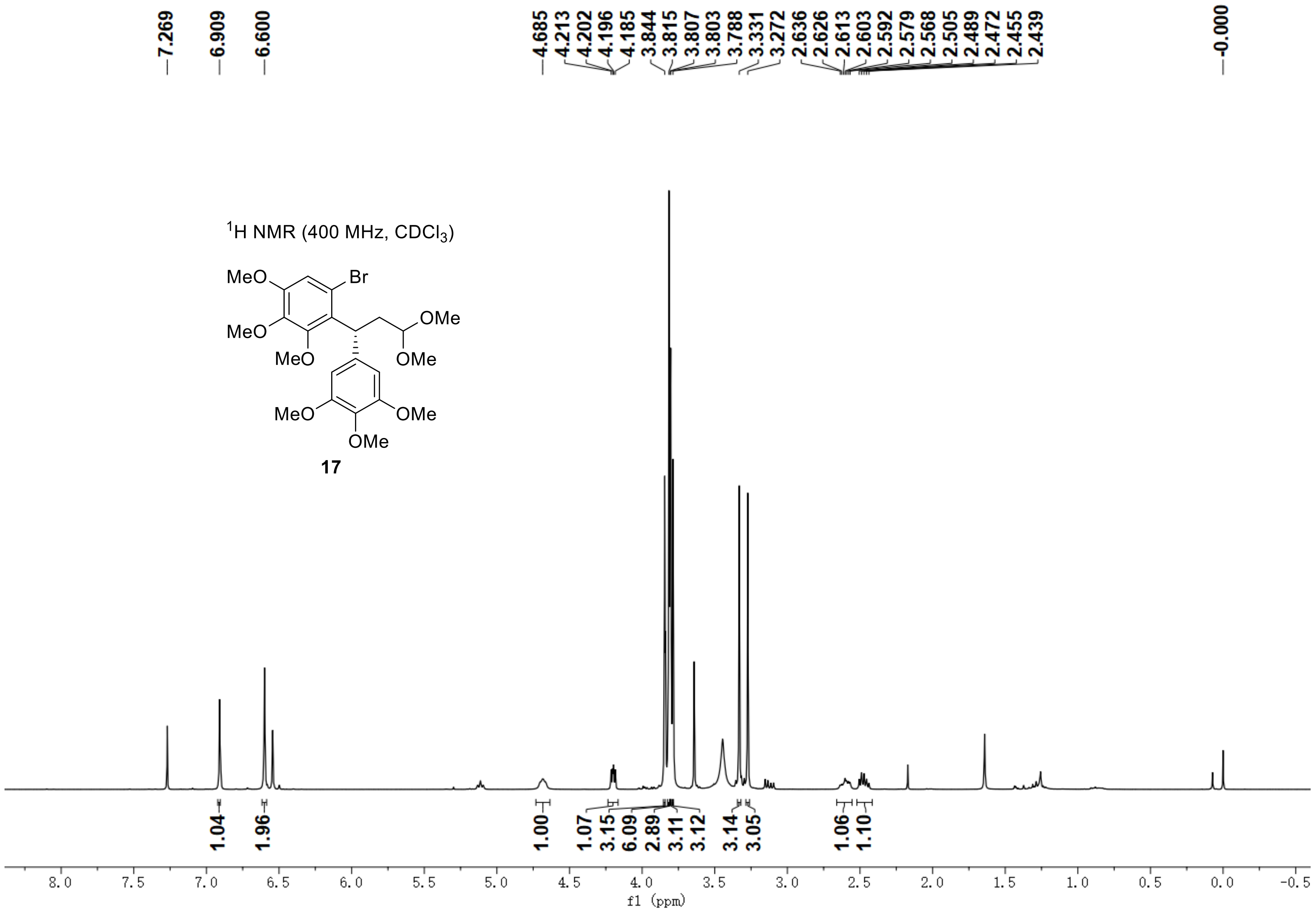

^{1}H NMR (400 MHz, $CDCl_3$)
MeO
Br
MeO
OMe
MeO
OMe
MeO
OMe
OMe
17
7.269
6.909
6.600
4.685
4.213
4.202
4.196
4.185
3.844
3.815
3.807
3.803
3.788
3.331
3.272
2.636
2.626
2.613
2.603
2.592
2.579
2.568
2.505
2.489
2.472
2.455
2.439
-0.000
1.04
1.96
1.00
1.07
3.15
6.09
2.89
3.11
3.12
3.14
3.05
1.06
1.10
8.0 7.5 7.0 6.5 6.0 5.5 5.0 4.5 4.0 3.5 3.0 2.5 2.0 1.5 1.0 0.5 0.0 -0.5
f1 (ppm)

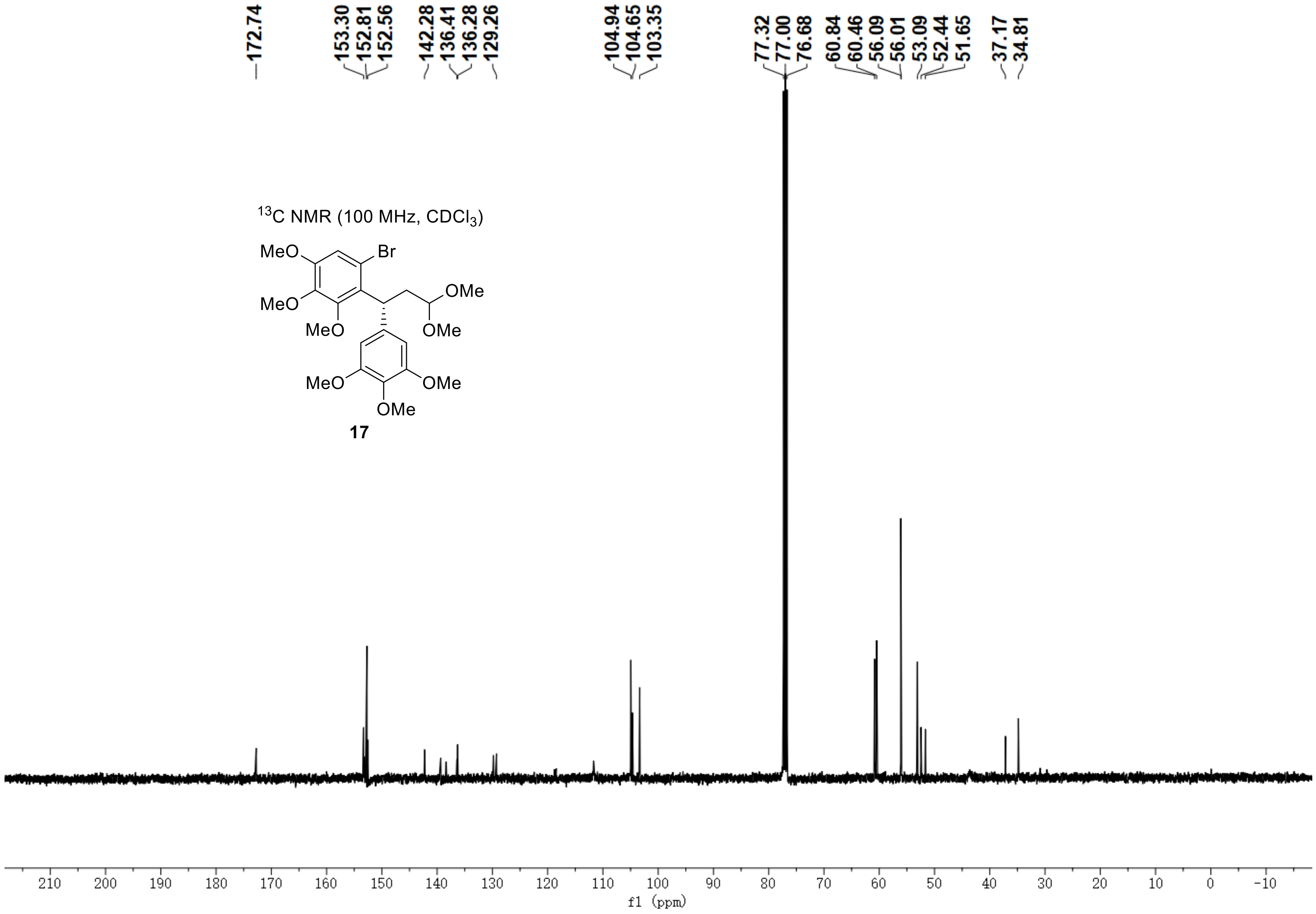
172.74
153.30
152.81
152.56
142.28
136.41
136.28
129.26
104.94
104.65
103.35
77.32
77.00
76.68
60.84
60.46
56.09
56.01
53.09
52.44
51.65
37.17
34.81
^{13}C NMR (100 MHz, CDCl$_3$)
MeO
Br
MeO
OMe
MeO
OMe
MeO
OMe
OMe
17
210 200 190 180 170 160 150 140 130 120 110 100 90 80 70 60 50 40 30 20 10 0 -10
f1 (ppm)

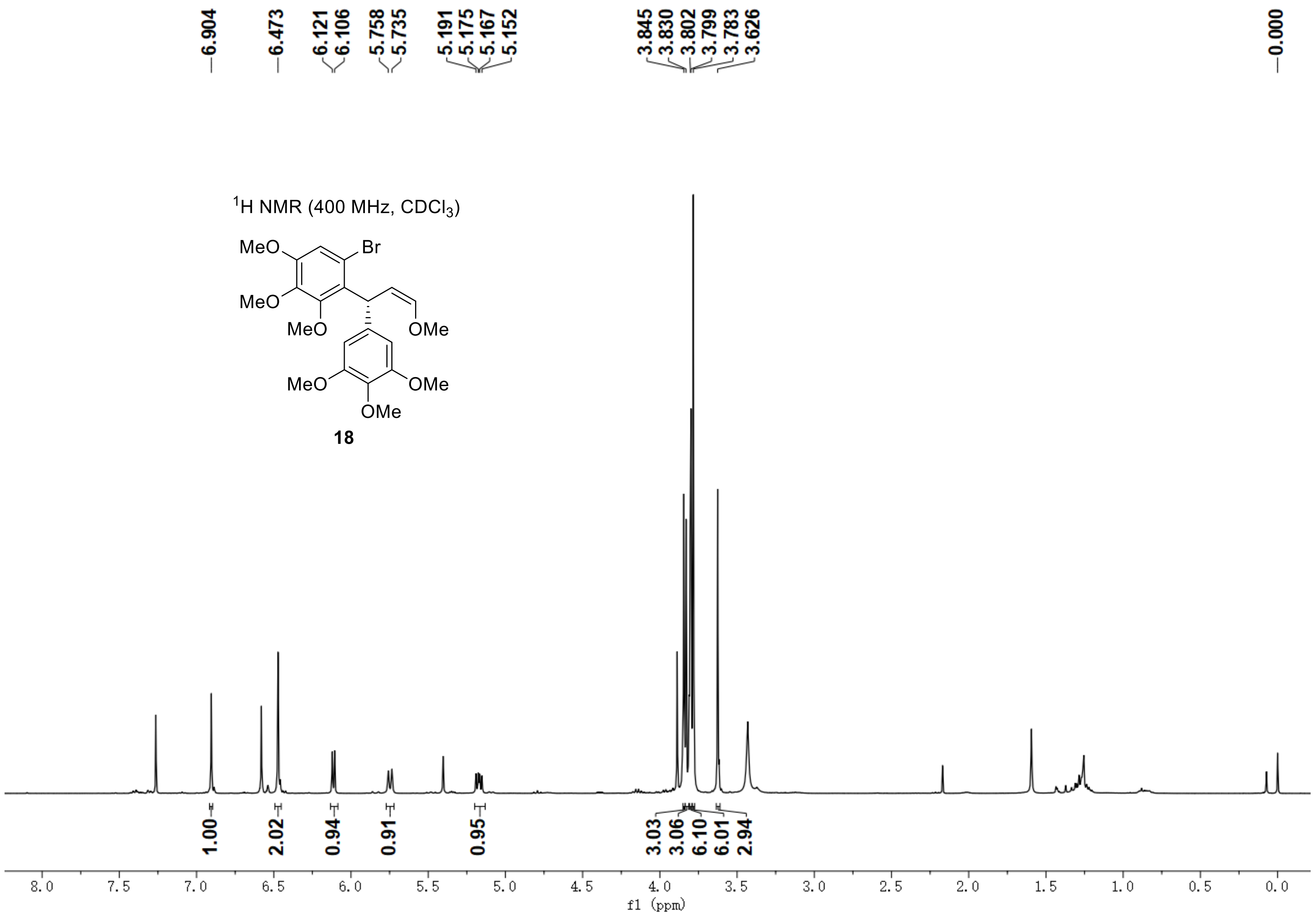
6.904
6.473
6.121
6.106
5.758
5.735
5.191
5.175
5.167
5.152
3.845
3.830
3.802
3.799
3.783
3.626
0.000
1H NMR (400 MHz, CDCl3)
MeO
Br
MeO
MeO
OMe
MeO
OMe
OMe
18
1.00
2.02
0.94
0.91
0.95
3.03
3.06
6.10
6.01
2.94
8.0
7.5
7.0
6.5
6.0
5.5
5.0
4.5
4.0
3.5
3.0
2.5
2.0
1.5
1.0
0.5
0.0
f1 (ppm)

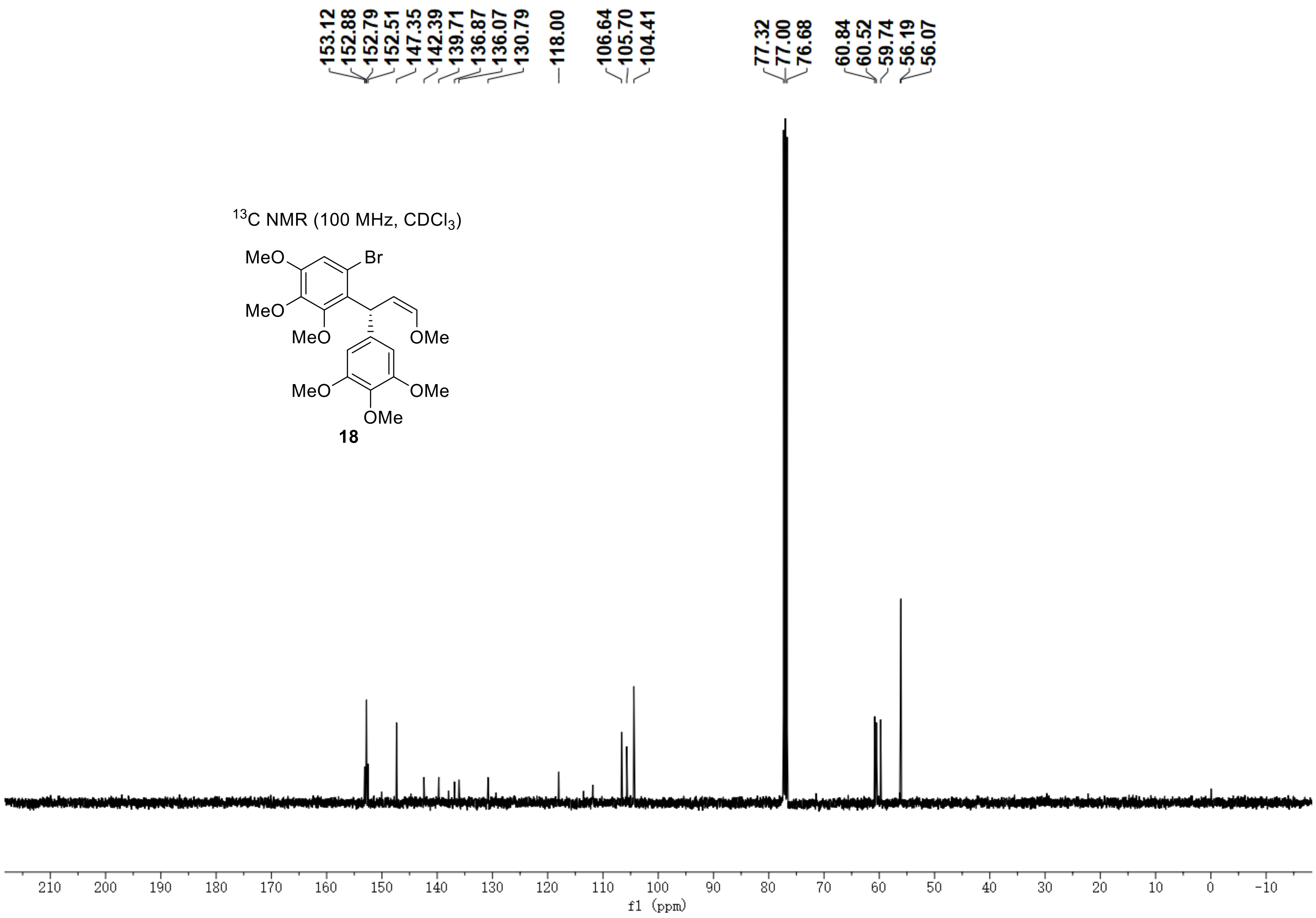
153.12
152.88
152.79
152.51
147.35
142.39
139.71
136.87
136.07
130.79
118.00
106.64
105.70
104.41
77.32
77.00
76.68
60.84
60.52
59.74
56.19
56.07
^{13}C NMR (100 MHz, CDCl$_3$)
MeO
Br
MeO
MeO
OMe
MeO
OMe
OMe
18
210 200 190 180 170 160 150 140 130 120 110 100 90 80 70 60 50 40 30 20 10 0 -10
f1 (ppm)

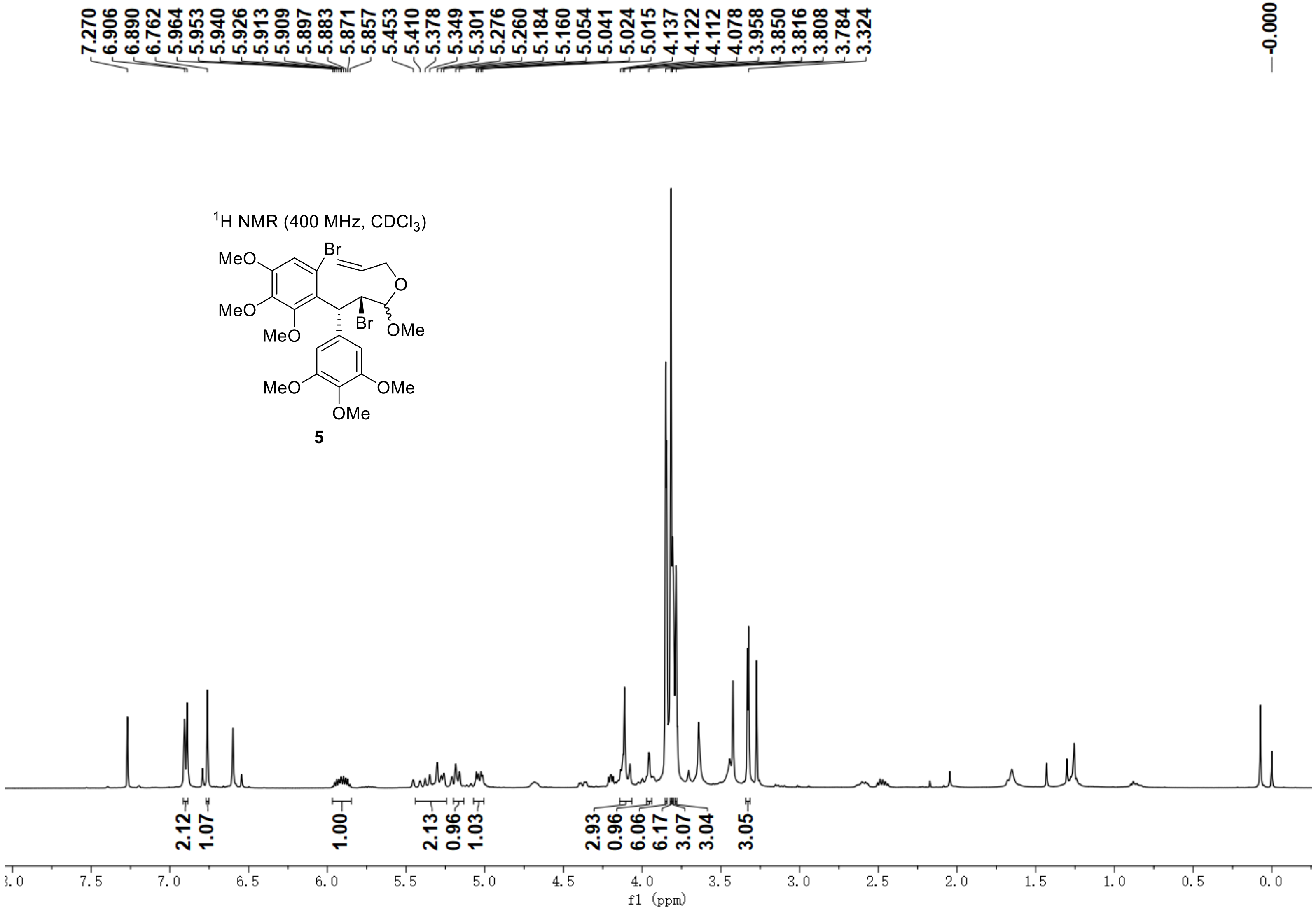

7.270 6.906 6.890 6.762 5.964 5.953 5.940 5.926 5.913 5.909 5.897 5.883 5.871 5.857 5.453 5.410 5.378 5.349 5.301 5.276 5.260 5.184 5.160 5.054 5.041 5.024 5.015 4.137 4.122 4.112 4.078 3.958 3.850 3.816 3.808 3.784 3.324
-0.000
^{1}H NMR (400 MHz, CDCl$_3$)
MeO
MeO
MeO
Br
O
Br
OMe
MeO
OMe
OMe
5
2.12 1.07 1.00 2.13 0.96 1.03 2.93 0.96 6.06 6.17 3.07 3.04 3.05
8.0 7.5 7.0 6.5 6.0 5.5 5.0 4.5 4.0 3.5 3.0 2.5 2.0 1.5 1.0 0.5 0.0
f1 (ppm)

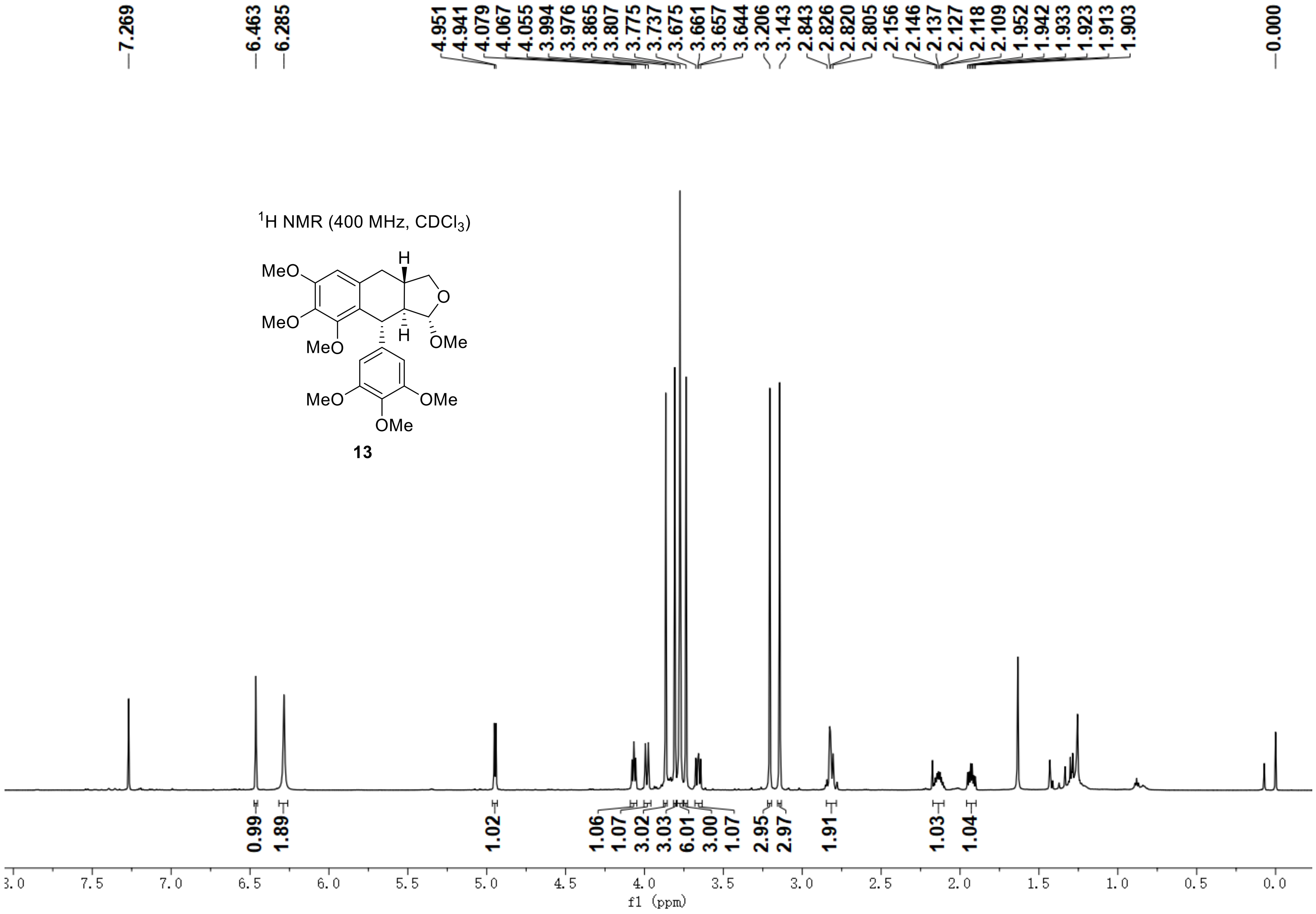

7.269
6.463
6.285
4.951
4.941
4.079
4.067
4.055
3.994
3.976
3.865
3.807
3.775
3.737
3.675
3.661
3.657
3.644
3.206
3.143
2.843
2.826
2.820
2.805
2.156
2.146
2.137
2.127
2.118
2.109
1.952
1.942
1.933
1.923
1.913
1.903
0.000
^{1}H NMR (400 MHz, $CDCl_3$)
MeO
MeO
MeO
H
H
O
OMe
MeO
OMe
OMe
13
0.99
1.89
1.02
1.06
1.07
3.02
3.03
6.01
3.00
1.07
2.95
2.97
1.91
1.03
1.04
8.0
7.5
7.0
6.5
6.0
5.5
5.0
4.5
4.0
3.5
3.0
2.5
2.0
1.5
1.0
0.5
0.0
f1 (ppm)

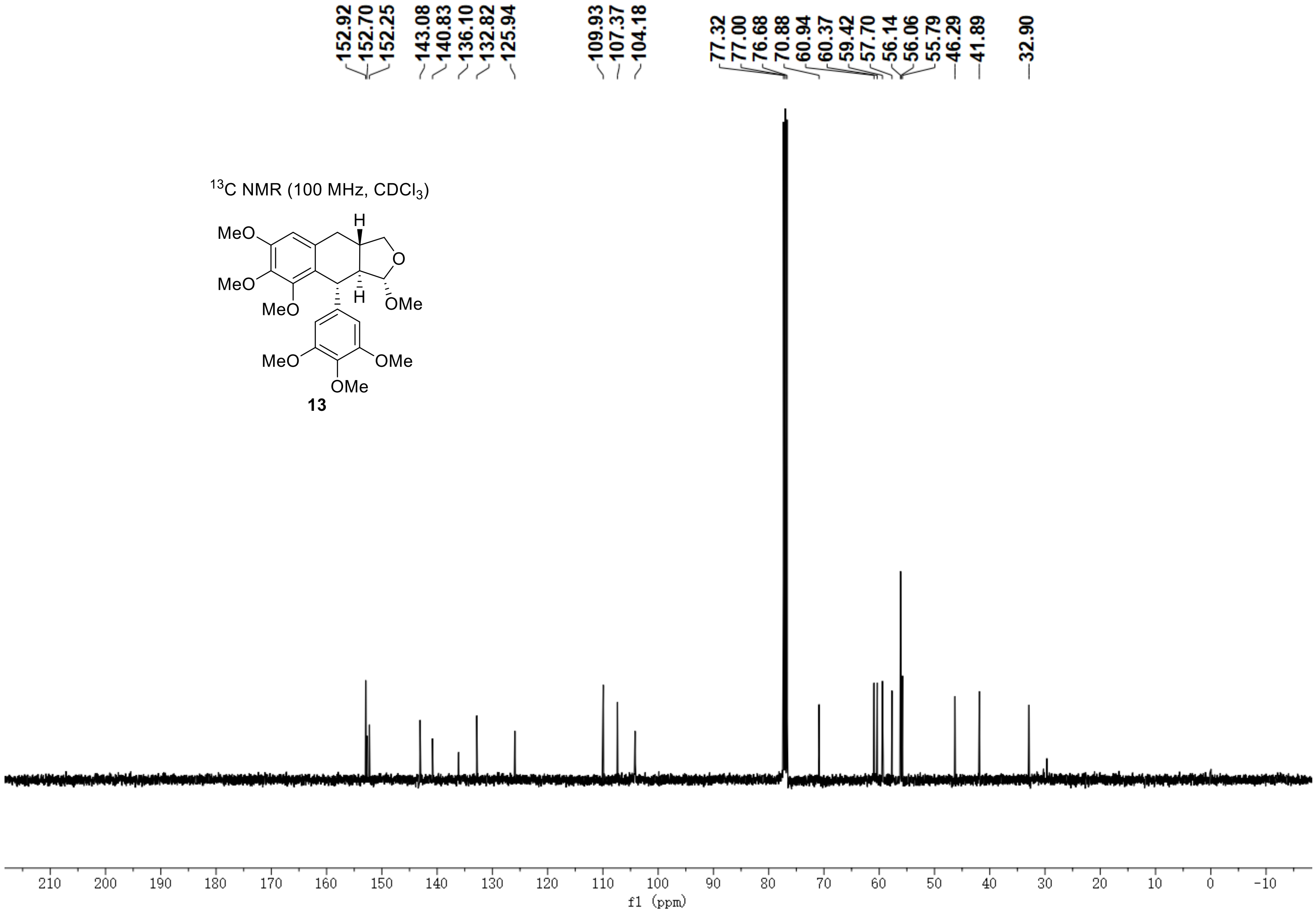
152.92
152.70
152.25
143.08
140.83
136.10
132.82
125.94
109.93
107.37
104.18
77.32
77.00
76.68
70.88
60.94
60.37
59.42
57.70
56.14
56.06
55.79
46.29
41.89
32.90
^{13}C NMR (100 MHz, $CDCl_3$)
MeO
MeO
MeO
H
H
O
OMe
MeO
OMe
OMe
13
210 200 190 180 170 160 150 140 130 120 110 100 90 80 70 60 50 40 30 20 10 0 -10
f1 (ppm)

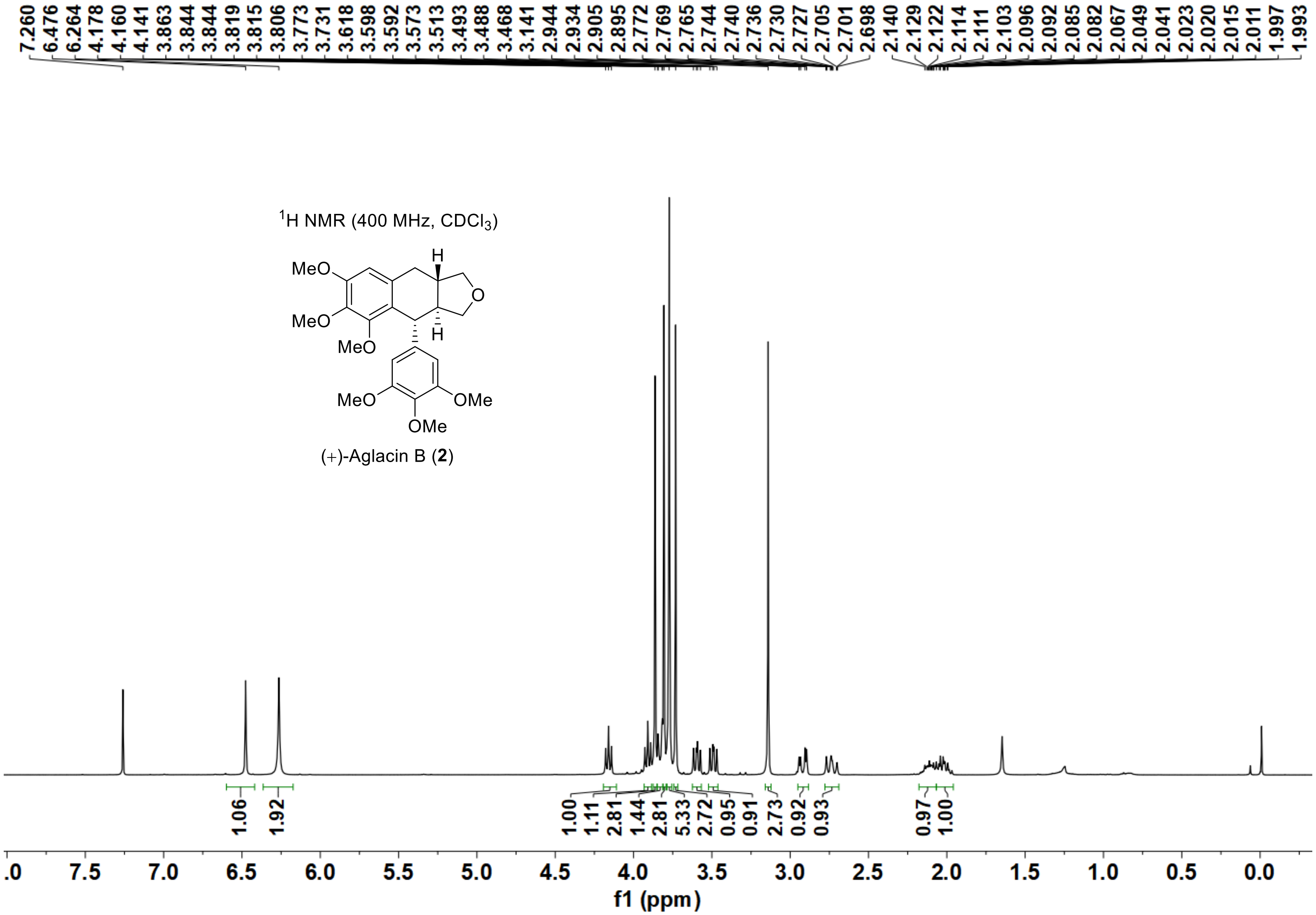
7.260 6.476 6.264 4.178 4.160 4.141 3.863 3.844 3.844 3.819 3.815 3.806 3.773 3.731 3.618 3.598 3.592 3.573 3.513 3.493 3.488 3.468 3.141 2.944 2.934 2.905 2.895 2.772 2.769 2.765 2.744 2.740 2.736 2.730 2.727 2.705 2.701 2.698 2.140 2.129 2.122 2.114 2.111 2.103 2.096 2.092 2.085 2.082 2.067 2.049 2.041 2.023 2.020 2.015 2.011 1.997 1.993
1H NMR (400 MHz, CDCl3)
MeO
MeO
MeO
H
H
O
MeO
OMe
OMe
(+)-Aglacin B (2)
1.06 1.92 1.00 1.11 2.81 1.44 2.81 5.33 2.72 0.95 0.91 2.73 0.92 0.93 0.97 1.00
.0 7.5 7.0 6.5 6.0 5.5 5.0 4.5 4.0 3.5 3.0 2.5 2.0 1.5 1.0 0.5 0.0
f1 (ppm)

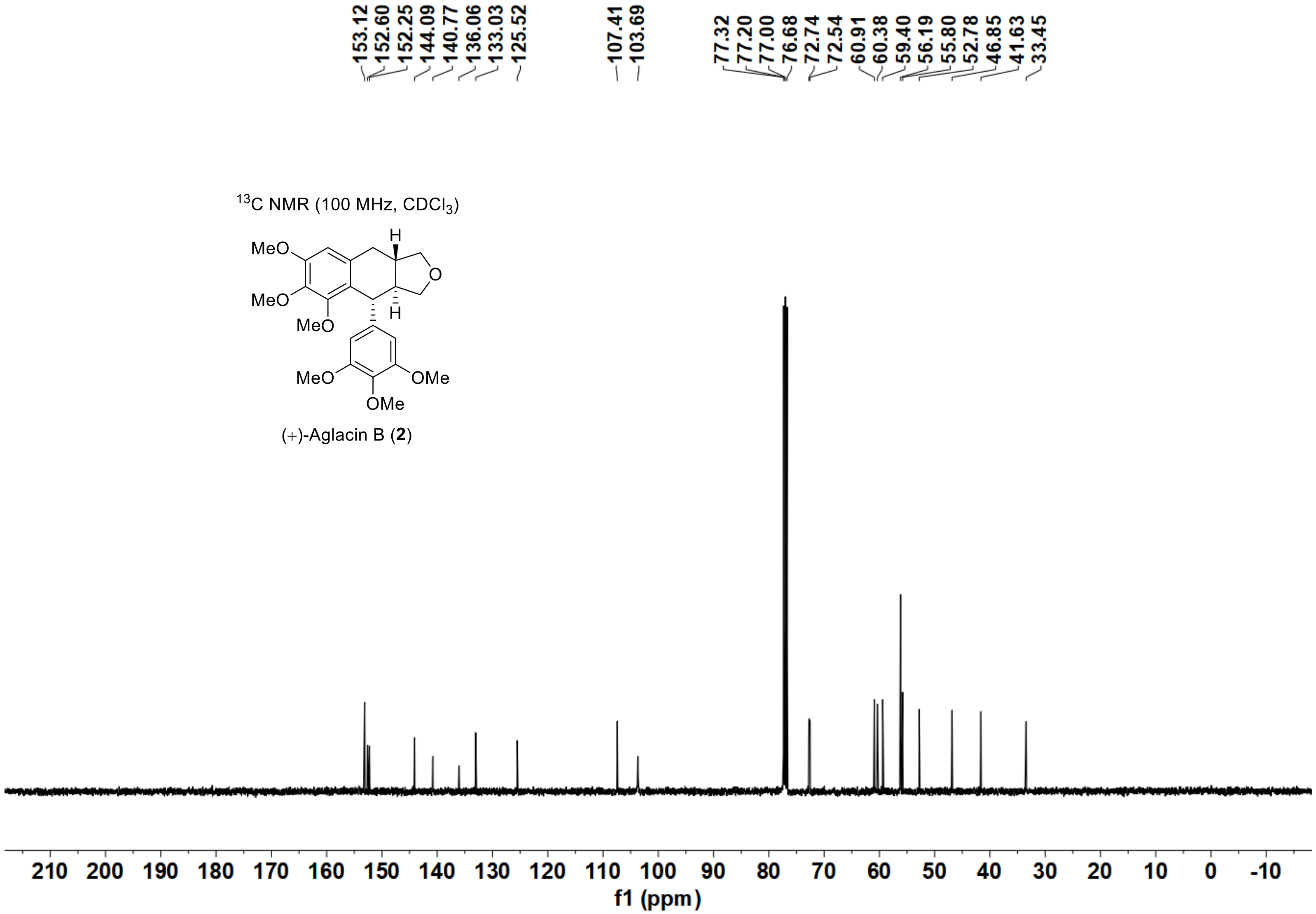
153.12
152.60
152.25
144.09
140.77
136.06
133.03
125.52
107.41
103.69
77.32
77.20
77.00
76.68
72.74
72.54
60.91
60.38
59.40
56.19
55.80
52.78
46.85
41.63
33.45
^{13}C NMR (100 MHz, $CDCl_3$)
MeO
MeO
MeO
H
H
O
MeO
OMe
OMe
210 200 190 180 170 160 150 140 130 120 110 100 90 80 70 60 50 40 30 20 10 0 -10
f1 (ppm)
(+)-Aglacin B (**2**)
